# Posicionamento sobre Diagnóstico e Tratamento da Tempestade Elétrica – 2026

**DOI:** 10.36660/abc.20260215

**Published:** 2026-04-28

**Authors:** Alexandre de Matos Soeiro, Cristiano Faria Pisani, João Luiz Fernandes Petriz, Bruno Ferraz de Oliveira Gomes, Cláudio Bittencourt das Virgens, José Roberto de Oliveira Silva, Paulo Rogério Soares, Tatiana de Carvalho Andreucci Torres Leal, Alexsandro Alves Fagundes, Luciana Vidal Armaganijan, Ana Luiza Moreno, Francisco Carlos da Costa Darrieux, Luciana Sacilotto, Maurício Pimentel, Fátima Dumas Cintra

**Affiliations:** 1 Universidade de São Paulo nstituto do Coração Hospital das Clínicas São Paulo SP Brasil Instituto do Coração do Hospital das Clínicas da Faculdade de Medicina da Universidade de São Paulo, São Paulo, SP – Brasil; 2 Universidade de São Paulo Hospital das Clínicas Faculdade de Medicina São Paulo SP Brasil Hospital das Clínicas da Faculdade de Medicina da Universidade de São Paulo, São Paulo, SP – Brasil; 3 Hospital Barra D’Or Rio de Janeiro RJ Brasil Hospital Barra D’Or, Rio de Janeiro, RJ – Brasil; 4 Universidade Federal da Bahia Hospital Universitário Professor Edgard Santos Salvador BA Brasil Hospital Universitário Professor Edgard Santos da Universidade Federal da Bahia, Salvador, BA – Brasil; 5 Universidade do Estado da Bahia Salvador BA Brasil Universidade do Estado da Bahia, Salvador, BA – Brasil; 6 Hospital Sírio Libanês São Paulo SP Brasil Hospital Sírio Libanês, São Paulo, SP – Brasil; 7 Universidade do Estado da Bahia Salvador BA Brasil Universidade do Estado da Bahia, Salvador, BA – Brasil; 8 Instituto Dante Pazzanese de Cardiologia São Paulo SP Brasil Instituto Dante Pazzanese de Cardiologia, São Paulo, SP – Brasil; 9 Instituto D’Or de Pesquisa e Ensino Rio de Janeiro RJ Brasil Instituto D’Or de Pesquisa e Ensino, Rio de Janeiro, RJ – Brasil; 10 Hospital de Clínicas de Porto Alegre Porto Alegre RS Brasil Hospital de Clínicas de Porto Alegre, Porto Alegre, RS – Brasil; 11 Universidade Federal de São Paulo São Paulo SP Brasil Universidade Federal de São Paulo, São Paulo, SP – Brasil

**Table:** 

Posicionamento sobre Diagnóstico e Tratamento da Tempestade Elétrica – 2026	
O relatório abaixo lista as declarações de interesse conforme relatadas à SBC pelos especialistas durante o período de desenvolvimento deste posicionamento, 2025/2026.	
Especialista	Tipo de relacionamento com a indústria
Alexandre de Matos Soeiro	Declaração financeira B - Financiamento de pesquisas sob sua responsabilidade direta/pessoal (direcionado ao departamento ou instituição) provenientes da indústria farmacêutica, de órteses, próteses, equipamentos e implantes, brasileiras ou estrangeiras: - Biomerieux.
Alexsandro Alves Fagundes	Nada a ser declarado
Ana Luiza Moreno de Almeida Silva	Nada a ser declarado
Bruno Ferraz de Oliveira Gomes	Nada a ser declarado
Claudio Marcelo Bittencourt das Virgens	Nada a ser declarado
Cristiano Faria Pisani	Declaração financeira A - Pagamento de qualquer espécie e desde que economicamente apreciáveis, feitos a (i) você, (ii) ao seu cônjuge/ companheiro ou a qualquer outro membro que resida com você, (iii) a qualquer pessoa jurídica em que qualquer destes seja controlador, sócio, acionista ou participante, de forma direta ou indireta, recebimento por palestras, aulas, atuação como proctor de treinamentos, remunerações, honorários pagos por participações em conselhos consultivos, de investigadores, ou outros comitês, etc. Provenientes da indústria farmacêutica, de órteses, próteses, equipamentos e implantes, brasileiras ou estrangeiras: - Johnson & Johnson.
Fatima Dumas Cintra	Outros relacionamentos Participação societária de qualquer natureza e qualquer valor economicamente apreciável de empresas na área de saúde, de ensino ou em empresas concorrentes ou fornecedoras da SBC: - Área da saúde.
Francisco Carlos da Costa Darrieux	Nada a ser declarado
João Luiz Fernandes Petriz	Declaração financeira A - Pagamento de qualquer espécie e desde que economicamente apreciáveis, feitos a (i) você, (ii) ao seu cônjuge/ companheiro ou a qualquer outro membro que resida com você, (iii) a qualquer pessoa jurídica em que qualquer destes seja controlador, sócio, acionista ou participante, de forma direta ou indireta, recebimento por palestras, aulas, atuação como proctor de treinamentos, remunerações, honorários pagos por participações em conselhos consultivos, de investigadores, ou outros comitês, etc. Provenientes da indústria farmacêutica, de órteses, próteses, equipamentos e implantes, brasileiras ou estrangeiras: - Servier: Vastarel.
José Roberto de Oliveira Silva Filho	Declaração financeira A - Pagamento de qualquer espécie e desde que economicamente apreciáveis, feitos a (i) você, (ii) ao seu cônjuge/ companheiro ou a qualquer outro membro que resida com você, (iii) a qualquer pessoa jurídica em que qualquer destes seja controlador, sócio, acionista ou participante, de forma direta ou indireta, recebimento por palestras, aulas, atuação como proctor de treinamentos, remunerações, honorários pagos por participações em conselhos consultivos, de investigadores, ou outros comitês, etc. Provenientes da indústria farmacêutica, de órteses, próteses, equipamentos e implantes, brasileiras ou estrangeiras: - Chiesi: Trimbow; GSK: Vacinas; Libbs: ticagrelor, ramipril e candesartana; Biolab: vasopressina. Outros relacionamentos Financiamento de atividades de educação médica continuada, incluindo viagens, hospedagens e inscrições para congressos e cursos, provenientes da indústria farmacêutica, de órteses, próteses, equipamentos e implantes, brasileiras ou estrangeiras: - Daiichi Sankyo: prasugrel.
Luciana Armaganijan	Nada a ser declarado
Luciana Sacilotto	Nada a ser declarado
Maurício Pimentel	Declaração financeira A - Pagamento de qualquer espécie e desde que economicamente apreciáveis, feitos a (i) você, (ii) ao seu cônjuge/ companheiro ou a qualquer outro membro que resida com você, (iii) a qualquer pessoa jurídica em que qualquer destes seja controlador, sócio, acionista ou participante, de forma direta ou indireta, recebimento por palestras, aulas, atuação como proctor de treinamentos, remunerações, honorários pagos por participações em conselhos consultivos, de investigadores, ou outros comitês, etc. Provenientes da indústria farmacêutica, de órteses, próteses, equipamentos e implantes, brasileiras ou estrangeiras: - Libbs: fibrilação atrial.
Paulo Rogério Soares	Nada a ser declarado
Tatiana de Carvalho Andreuci Torres Leal	Declaração financeira Outros relacionamentos Financiamento de atividades de educação médica continuada, incluindo viagens, hospedagens e inscrições para congressos e cursos, provenientes da indústria farmacêutica, de órteses, próteses, equipamentos e implantes, brasileiras ou estrangeiras: - Lilly: Mounjaro.

## Sumário

**1. Introdução** 4
  **1.1. Objetivos do Posicionamento** 4
  **1.2. Métodos** 4
**2. Fisiopatologia** 5
**3. Abordagem Inicial na Sala de Emergência** 6
  **3.1. Diagnóstico** 6
  **3.2. Eletrocardiograma** 7
  **3.3. Exames Laboratoriais e Bioquímica** 7
  **3.4. Avaliação do Cardioversor-Desfibrilador Implantável** 7
  **3.5. Passos Iniciais da Estabilização Inicial** 7
**4. Terapia Clínica Medicamentosa** 8
  **4.1. Antiarrítmicos** 9
  **4.2. Amiodarona** 9
  **4.3. Lidocaína** 10
  **4.4. Betabloqueadores Adrenérgicos** 10
  **4.5. Sulfato de Magnésio** 11
**5. Manejo e Programação do Cardioversor-Desfibrilador Implantável** 11
  **5.1. Terapias do Cardioversor-Desfibrilador Implantável** 11
  **5.2. Detecção de Eventos** 11
  **5.3. Choque** 13
  **5.4. Terapia Antitaquicardia** 13
  **5.5. Estimulação Cardíaca Artificial** 13
  **5.6. Abordagem do Paciente com Cardioversor-Desfibrilador Implantável em Tempestade Elétrica** 13
**6. Ablação por Cateter no Contexto de Tempestade Elétrica** 15
  **6.1. Terapias Adicionais para Tempestade Elétrica** 16
    **6.1.1. Sedação** 16
    **6.1.2. Anestesia Torácico-Epidural** 17
    **6.1.3. Bloqueio do Gânglio Estrelado** 17
    **6.1.4. Denervação Simpática Cardíaca Cirúrgica** 17
    **6.1.5. Denervação Simpática Renal** 18
    **6.1.6. Manejo Circulatório** 18
    **6.1.7. Oxigenação por Membrana Extracorpórea** 18
    **6.1.8. Balão Intra-Aórtico** 18
    **6.1.9. Suporte Circulatório TandemHeart e Dispositivo Impella** 18
    **6.1.10. Escolha do Dispositivo** 18
**7. Manejo da Tempestade Elétrica Associada a Canalopatias** 18
**8. Prevenção da Recorrência a Longo Prazo** 21
  **8.1. Terapia Farmacológica** 21
  **8.2. Cardioversor-Desfibrilador Implantável** 21
  **8.3. Ablação por Cateter** 22
  **8.4. Modulação Autonômica** 22
**9. Novas Perspectivas** 22
**Referências** 22

## 1. Introdução

A tempestade elétrica (TE) é definida como uma condição de instabilidade elétrica cardíaca caracterizada pela ocorrência de três ou mais episódios de taquicardia ventricular (TV) sustentada, fibrilação ventricular (FV) ou choques apropriados do cardioversor-desfibrilador implantável (CDI) em um período de 24 horas, sendo esses episódios separados por pelo menos 5 minutos (Figura Central).^1,2^

**Figure:**
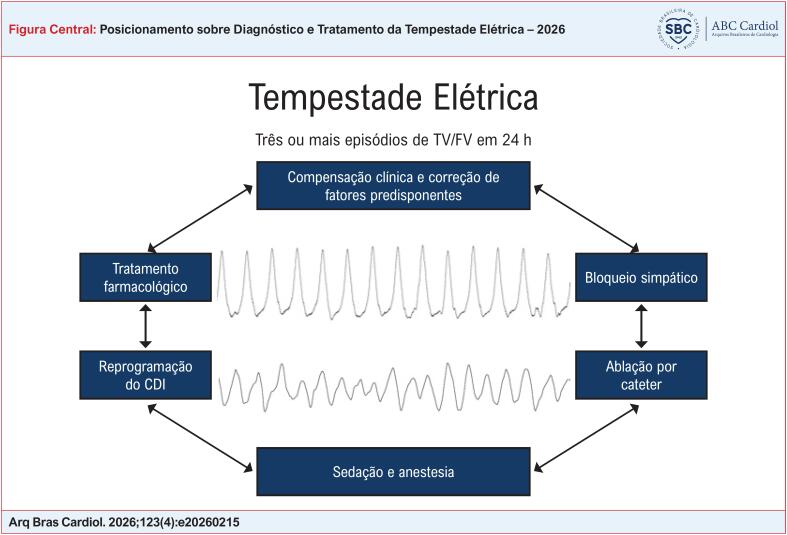


A TE ocorre em 4 a 40% dos pacientes com CDI, com uma mediana de dois episódios por paciente e aumento da incidência nos primeiros dias após o implante do dispositivo. Pacientes que, além de arritmia ventricular, também apresentam episódios repetitivos, sejam eles definidos como TE ou não, apresentam maior mortalidade, proporcional ao número de episódios em um menor período.^3^

A incidência de TE é menor nos pacientes em prevenção primária. Em uma subanálise com 719 pacientes do estudo *Multileft Automatic Defibrillator Implantation Trial II* (MADIT-II), que avaliou a indicação de CDI para profilaxia primária em pacientes com infarto agudo do miocárdio (IAM) prévio e fração de ejeção do ventrículo esquerdo (FEVE) ≤ 30%, a incidência de TE foi de 4,4% em um seguimento médio de 20,6 meses.^4-8^ Além da presença do CDI, outros fatores de risco são comumente encontrados em pacientes com TE na emergência, como fração de ejeção reduzida, doença renal crônica e IAM prévio.^6^

Em uma metanálise publicada recentemente, a incidência de TE foi de 9,1% ao ano em pacientes com cardiopatia chagásica crônica.^9-11^ Em outro estudo, a ocorrência de choques repetitivos, principalmente acima de quatro por mês, foi associada a um aumento na mortalidade de pacientes com cardiopatia chagásica crônica, sendo a expectativa de vida média desses pacientes de 2,1 meses.^12^

A TE é uma condição clínica associada a mortalidade elevada, que pode chegar até 14% nas primeiras 48 horas. Mesmo em comparação com pacientes que apresentam episódios isolados de TV/FV, a TE aumenta em 2,4 vezes o risco de morte na internação e em até 5,4 vezes nos 3 meses consecutivos. Portanto, é imprescindível que os médicos clínicos, cardiologistas, emergencistas e intensivistas reconheçam e tratem adequadamente a TE.^13^

### 1.1. Objetivos do Posicionamento

- Fornecer orientação prática para médicos (cardiologistas ou não) e equipes multiprofissionais sobre a abordagem inicial, o diagnóstico e a estabilização clínica de pacientes com TE, de acordo com as melhores práticas baseadas em evidência;
- Estabelecer a rotina de tratamento clínico medicamentoso para pacientes em TE, conforme as evidências e a realidade do cenário nacional;
- Relatar as indicações e os cenários para utilização de ablação por cateter com terapia adicional;
- Discutir, à luz das evidências, terapias adicionais relevantes, suas indicações e perspectivas a longo prazo.

### 1.2. Métodos

Recomendações:

- **Classe I:** condições para as quais há evidências conclusivas ou, na sua falta, consenso geral de que o procedimento é seguro e útil/eficaz;
- **Classe II:** condições para as quais há evidências conflitantes e/ou divergência de opinião sobre a segurança e utilidade/eficácia do procedimento;
- **Classe IIa:** peso ou evidência/opinião a favor do procedimento. A maioria aprova;
- **Classe IIb:** segurança e utilidade/eficácia menos bem estabelecidas, não havendo predomínio de opiniões a favor;
- **Classe III:** condições para as quais há evidências e/ou consenso de que o procedimento não é útil/eficaz e, em alguns casos, pode ser prejudicial.

Evidências:

- **Nível A:** dados obtidos a partir de múltiplos estudos randomizados de bom porte, concordantes e/ou de metanálise robusta de estudos clínicos randomizados;
- **Nível B:** dados obtidos a partir de metanálise menos robusta, a partir de um único estudo randomizado ou de estudos não randomizados (observacionais);
- **Nível C:** dados obtidos de opiniões consensuais de especialistas.

## 2. Fisiopatologia

**Figura 1:**
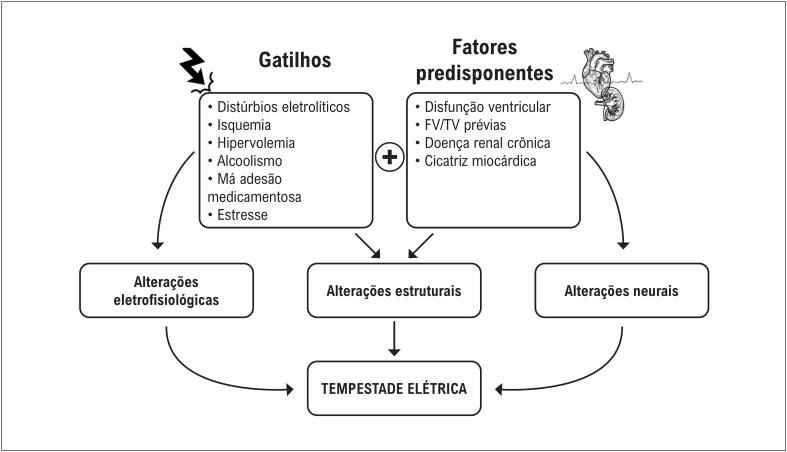
Contribuição dos substratos e mecanismos predisponentes para a fisiopatologia da geração da tempestade elétrica. Adaptado de Looi et al.^15^

A TE pode ocorrer em uma diversidade de situações, como IAM, cardiopatia isquêmica, cardiopatia não isquêmica e na presença de síndromes arrítmicas hereditárias (como síndrome de Brugada e síndrome do QT curto, entre outras).^14^ Quando é identificado um paciente com TE, deve-se investigar os potenciais gatilhos e substratos relacionados à sua ocorrência (Figura 1). Esses substratos e mecanismos estão diretamente envolvidos na fisiopatologia da TE.^15^

Para a ocorrência da TE, é necessária a interação entre o sistema nervoso autônomo e algum substrato eletrofisiológico predisponente. Essa interação varia ao longo do tempo em virtude de processos como cicatrização miocárdica, isquemia ou dilatação ventricular secundária à insuficiência cardíaca progressiva. Assim, o reconhecimento de gatilhos e preditores é fundamental para a identificação de pacientes sob maior risco, visando à redução do risco e da ocorrência da TE.

No entanto, na maioria dos casos, não é identificada uma causa clara para o desenvolvimento da TE. No estudo *Shock Inhibition Evaluation With Azimilide* (SHIELD), um gatilho para a TE foi identificado em somente 13% dos pacientes.^7^ Da mesma forma, fatores de risco para a TE podem ser difíceis de identificar. Em um estudo com pacientes usuários de CDI, as variáveis mais associadas à TE foram fração de ejeção reduzida e uso de medicamentos antiarrítmicos classe IA.^16^

Doenças estruturais cardíacas que podem predispor à ocorrência de TE incluem fibrose miocárdica, deposição de gordura ou, até mesmo, pequenas cicatrizes. Nesses casos, o substrato pode desencadear TV reentrante ou focal. Automaticidade anormal ou atividade trigada podem estar correlacionadas ao início e à manutenção da taquiarritmia. No entanto, os fatores específicos que determinam a TE não são plenamente conhecidos.

A TV monomórfica (TVM) é a forma mais comum de TE. Em uma metanálise envolvendo pacientes com TE, 94% apresentavam alguma cardiopatia estrutural (isquêmica ou não isquêmica), sendo que 80% dos casos tratavam-se de TVM com mecanismo relacionado à cicatriz miocárdica.^17^ Em contrapartida, a TV polimórfica (TVP) e a FV estão mais frequentemente associadas ao cenário de IAM e insuficiência cardíaca avançada.

O sistema nervoso autônomo possui um papel fundamental na modulação da arritmogênese. No contexto da TE, o tônus simpático encontra-se aumentado em pacientes com cardiopatia estrutural. No estudo *Temperature-Related Incidence of Electrical Storm* (TEMPEST), em que 83% dos pacientes possuíam alguma cardiomiopatia, a prevalência de TE foi maior nos dias de semana e durante o período de trabalho.^18^ Em algumas cardiopatias hereditárias, como a síndrome do QT longo (SQTL) e a TV catecolaminérgica, os eventos arrítmicos são desencadeados por estresse físico ou emocional.

Alguns mecanismos moleculares foram propostos na arritmogênese da TE. O principal deles envolve alterações na fosforilação de proteínas que regulam o cálcio, promovendo desequilíbrio na quantidade de cálcio no cardiomiócito e levando a gatilhos arritmogênicos mediados por pós-despolarização, hipocontratilidade resultante da depleção de cálcio no retículo sarcoplasmático e atividade trigada.

De fato, diversos mecanismos foram propostos, mas ainda existem poucas certezas quanto ao real entendimento de toda a fisiopatologia da TE. Novos estudos são necessários para compreender melhor essa entidade.

## 3. Abordagem Inicial na Sala de Emergência

### 3.1. Diagnóstico

A avaliação inicial na sala de emergência começa pelo reconhecimento do paciente com risco de apresentar TE. A maioria dos pacientes com TE atendidos na emergência apresenta alguma doença cardíaca estrutural, como insuficiência cardíaca com FEVE reduzida (de etiologia isquêmica ou não isquêmica), cardiopatia arritmogênica do ventrículo direito, sarcoidose, amiloidose, doença de Chagas e canalopatias, como a síndrome de Brugada.^19,20^

O primeiro passo na avaliação do paciente com TE é identificar a presença de instabilidade hemodinâmica; quando presente, deve ser manejada conforme as diretrizes de Suporte Avançado de Vida em Cardiologia (ACLS).^21,22^ Pacientes que se apresentam com arritmias ventriculares e sem pulso devem ser rapidamente desfibrilados. Distúrbios hidroeletrolíticos devem ser corrigidos, com atenção especial à hipomagnesemia e hipopotassemia. A equipe de hemodinâmica deve ser acionada para o encaminhamento do paciente quando houver suspeita de isquemia miocárdica, principalmente na presença de supradesnivelamento do segmento ST. Nos casos em que a TE decorre de descompensação da insuficiência cardíaca, deve-se instituir suporte hemodinâmico com medicamentos vasoativos e, se necessário, dispositivos de assistência ventricular. Esses pacientes devem ser encaminhados para unidades de terapia intensiva para seguimento e manejo das complicações do quadro.

Após a estabilização inicial do paciente, é necessário investigar potenciais causas reversíveis de TE, como toxicidade medicamentosa, descompensação da insuficiência cardíaca, nova isquemia miocárdica, tireotoxicose, infecções, febre e distúrbios hidroeletrolíticos, em especial hipocalemia e hipomagnesemia.^4^ Deve-se questionar sobre o uso de medicações que prolongam o aumento do intervalo QT, incluindo antiarrítmicos, que, embora úteis em diversas situações, também podem aumentar o intervalo QT, como a amiodarona.^16^

O quadro clínico de um episódio de TE pode ser bastante variável, incluindo palpitações, dor torácica, síncope, descompensação da insuficiência cardíaca, hipotensão e parada cardíaca.^18,23^ A apresentação depende de diversos fatores, como a frequência cardíaca, o grau de disfunção ventricular, a doença de base e a presença de CDI. Pacientes com disfunção ventricular mais significativa podem não tolerar episódios de TE. Em algumas situações, os pacientes podem apresentar maior estabilidade, com frequência cardíaca mais baixa, configurando uma TV lenta, em torno de 100 a 120 batimentos por minuto.

### 3.2. Eletrocardiograma

Todos os pacientes com suspeita de TE devem realizar um eletrocardiograma (ECG) de 12 derivações e ser submetidos a monitoramento cardíaco contínuo.

A maioria dos episódios de TE manifesta-se pela presença de TVM, geralmente secundária a uma cicatriz, levando a um mecanismo de reentrada. Em contrapartida, a TVP e a FV são geralmente descritas em casos de isquemia aguda, distúrbios hidroeletrolíticos, aumento do intervalo QT e canalopatias genéticas.

Em algumas situações, a diferenciação entre taquicardia supraventricular com condução aberrante e TV pode ser desafiadora. Por esse motivo, diversos algoritmos foram elaborados nos últimos anos para auxiliar nessa etapa do diagnóstico das arritmias ventriculares.^24-26^ Em situações extremas, com instabilidade hemodinâmica e risco iminente de óbito, as taquicardias de QRS largo devem ser tratadas imediatamente como arritmias ventriculares.

### 3.3. Exames Laboratoriais e Bioquímica

A avaliação laboratorial deve incluir hemograma completo, troponina ultrassensível, peptídeo natriurético cerebral (BNP), hormônios tireoidianos (TSH e T4 livre), eletrólitos (sódio, potássio, cálcio e magnésio) e outros exames direcionados à possível etiologia, como função renal e proteína C reativa.

### 3.4. Avaliação do Cardioversor-Desfibrilador Implantável

Em portadores de CDI que receberam terapias do dispositivo, o primeiro passo após a estabilização clínica é determinar se a terapia foi adequada ou não. Terapias inadequadas podem acontecer em até 40% dos casos, mesmo com os avanços nos algoritmos de detecção de arritmias ventriculares dos dispositivos.^27^

Na avaliação do dispositivo, também é possível avaliar a quantidade e a data das arritmias ventriculares, a frequência cardíaca, a morfologia e o início da arritmia, a forma como o CDI respondeu a essas arritmias (por exemplo, terapia antitaquicardia [ATP] ou choque) e a resposta do paciente às terapias. É importante ressaltar que pacientes submetidos a choques frequentes pelo CDI apresentam maior risco de desenvolver transtornos psiquiátricos associados, como ansiedade e depressão.

As causas mais comuns de terapias inapropriadas são arritmias supraventriculares em zona de TV/FV (por exemplo, fibrilação atrial com alta resposta ventricular), *oversensing* do eletrodo ventricular (por exemplo, *oversensing* de onda T) e disfunção do eletrodo ventricular decorrente de fratura ou defeito de isolamento.^28^

A radiografia de tórax deve ser realizada em todos os pacientes com suspeita de terapia inapropriada pelo CDI, a fim de diagnosticar deslocamento ou fratura do eletrodo do dispositivo.

### 3.5. Passos Iniciais da Estabilização Inicial

A abordagem tradicional de taquiarritmias de QRS largo orientada pelo ACLS geralmente não é a mais adequada para pacientes com TE, devido a suas peculiaridades.^2 9^ Durante um episódio de TE, ocorre aumento do tônus simpático, que pode ser exacerbado por choques repetitivos do CDI, sendo, por vezes, necessário desativar as terapias do dispositivo e tentar outra abordagem. Esse aumento das catecolaminas e da ativação simpática torna o coração mais suscetível à isquemia e a novas arritmias, intensificando o estado de TE.^30^

Nesse contexto, a supressão do sistema nervoso simpático por meio do uso de betabloqueadores possui um papel fundamental no tratamento da TE. Um estudo que avaliou pacientes com infarto do miocárdio prévio e TE demonstrou que o uso de betabloqueadores reduziu a incidência de morte súbita.^31, 32^

#### 3.5.1. Betabloqueadores

Diversos estudos buscaram entender o papel dos betabloqueadores na TE e sua eficácia na redução do tônus simpático. Em estudos experimentais, o uso de betabloqueadores não seletivos reduziu o consumo de oxigênio miocárdico e aumentou sua oferta, reduzindo a injúria miocárdica e alterando os limiares elétricos para o desencadeamento de arritmias ventriculares.^33^ Diversos betabloqueadores já foram estudados, como o propranolol, um betabloqueador não seletivo, lipofílico e capaz de atravessar a barreira hematoencefálica, e betabloqueadores seletivos, como o esmolol, que pode ser titulado em infusão contínua.^34^ Um estudo clínico randomizado comparando propranolol na dose de 40 mg a cada 6 h com metoprolol mostrou maior eficácia do propranolol na estabilização da TE.^35^

#### 3.5.2. Amiodarona e Outros Antiarrítmicos

Embora outros antiarrítmicos tenham sido descritos para o tratamento da TE, a amiodarona permanece como o agente de primeira linha.^13,36^ Outros agentes, como os antiarrítmicos de classe I (por exemplo, lidocaína), não são recomendados como terapias de primeira linha, pois apresentam eficácia variável nesse cenário. A lidocaína pode ser uma opção em casos de TE secundária à isquemia miocárdica, embora seja inferior à amiodarona em pacientes com FV refratária.^37,38^ Em situações não relacionadas à isquemia miocárdica, a lidocaína apresenta baixa taxa de reversão de arritmias, com taxas de sucesso em torno de 8 a 30%.^39^

A terapia farmacológica para o tratamento da TE será abordada em tópico separado neste documento.

#### 3.5.3. Bloqueio Simpático

Durante o quadro de TE, os pacientes passam por intenso estresse físico e emocional, aumentando ainda mais a atividade do sistema nervoso simpático e perpetuando a ocorrência de arritmias ventriculares. Medidas para redução do estresse podem ser consideradas, como manter o paciente em ambiente calmo e utilizar doses baixas de ansiolíticos, como benzodiazepínicos.

Estudos sugerem uma redução da atividade do sistema nervoso simpático com o uso de bolus de sedação com propofol, devido à sua capacidade de supressão do sistema nervoso simpático.^40^ Em casos refratários, a intubação e a sedação profunda, mesmo com outros sedativos, como o midazolam, devem ser consideradas.

Outras opções menos invasivas incluem a anestesia peridural torácica, uma terapia alternativa para a inibição direta da inervação simpática cardíaca, sem os efeitos colaterais potenciais da sedação^41^, e o bloqueio do gânglio estrelado, que pode ser realizado por meio de punção paratraqueal guiada por ultrassonografia à beira do leito.^29,42^

#### 3.5.4. Manejo e Programação do Cardioversor-Desfibrilador Implantável

Em portadores de CDI, a verificação da ocorrência de terapias e se foram adequadas é um passo fundamental na avaliação inicial. Nos casos em que são detectadas terapias inadequadas, a desativação da terapia de choque do CDI deve preceder qualquer outra intervenção.^43^ Em situações em que o acesso aos programadores do dispositivo é limitado, a colocação de um ímã sobre o CDI pode ser utilizada para desativar as terapias de ATP e choques. As terapias inapropriadas geram um estado hiperadrenérgico que, por si só, pode precipitar a TE. Pelo mesmo motivo, mesmo quando as terapias são adequadas, é possível considerar a desativação temporária dos choques em pacientes hemodinamicamente estáveis.^28^

Em casos de choques apropriados em episódios de TE por TVM, podem ser realizados ajustes na programação de detecção e aprimoramento de terapias, sendo a ATP uma estratégia segura, eficaz e menos desconfortável para o paciente.^44,45^

Às vezes, os algoritmos de estimulação do dispositivo podem ter efeito pró-arritmogênicos. A programação e o manejo do CDI serão abordados em tópico específico neste documento.

#### 3.5.5. Situações Específicas

Por fim, existem duas situações em que o tratamento da TE apresenta exceções às regras gerais previamente descritas. A primeira refere-se aos pacientes com histórico de síndrome de Brugada ou alta suspeição com base em ECGs anteriores. A incidência de TE nessa população não é conhecida; contudo, trata-se de pacientes com risco elevado de desenvolver arritmias potencialmente fatais.^46^

Muitas das intervenções farmacológicas tradicionalmente utilizadas na TE não apresentam eficácia ou podem agravar ainda mais a TE em pacientes com síndrome de Brugada. Nessa população, o isoproterenol, um beta-agonista, deve ser considerado terapia de primeira linha, sendo também eficaz no tratamento de pacientes com TE associada à síndrome do QT curto.^47^

A segunda exceção ocorre quando o ritmo apresentado é TVP, que representa cerca de 7% dos casos de TE e pode ser o primeiro sinal de isquemia. Episódios recorrentes de TVP devem levantar a suspeita de síndrome do QT longo adquirida, cujo tratamento requer uma abordagem especial para evitar a degeneração em *torsades de pointes* (TdP).

Geralmente, o prolongamento adquirido do intervalo QT se deve à polifarmácia, com ou sem desequilíbrio eletrolítico, como hipocalemia e hipomagnesemia. Nesses casos, deve-se evitar o uso de amiodarona e suspender qualquer outro medicamento que possa causar prolongamento do intervalo QT, incluindo antibióticos. A correção de distúrbios hidroeletrolíticos deve ser realizada e, nos casos de arritmias relacionadas ao QT longo e à bradicardia, deve-se evitar a bradicardia, podendo-se até considerar a implantação de um marcapasso transvenoso para prevenção da bradicardia e realização *overdrive pacing*.^48^

**Figura 2:**
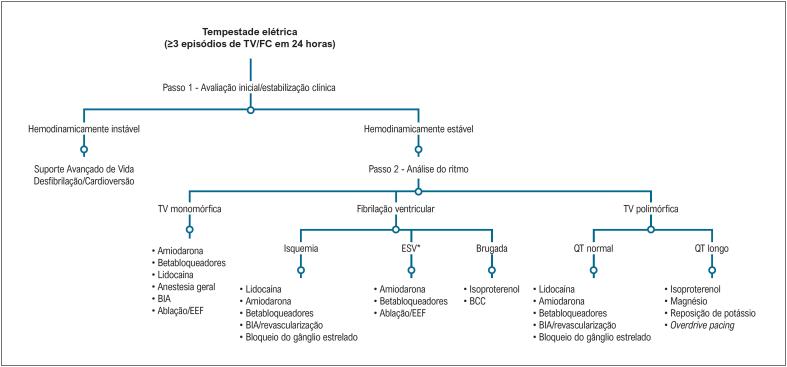
Algoritmo proposto para tratamento da tempestade elétrica. ESV: extrassístole ventricular induzindo tempestade elétrica; BIA: balão intra-aórtico, EEF: estudo eletrofisiológico; BCC: bloqueador de canal de cálcio. Adaptado de Eifling et al.^23^

A Figura 2 contém as principais abordagens listadas no manejo inicial da TE.

## 4. Terapia Clínica Medicamentosa

A terapia clínica medicamentosa baseia-se principalmente em quatro pilares: uso de antiarrítmicos, betabloqueadores, manejo da sedação e suporte hemodinâmico.

Vale ressaltar que a identificação de possíveis fatores desencadeadores, assim como sua resolução, faz parte do manejo clínico da TE. Isquemia miocárdica, insuficiência cardíaca descompensada, baixo débito e congestão pulmonar, tireotoxicose, distúrbios hidroeletrolíticos, toxicidade e uso de medicamentos pró-arrítmicos, assim como aumento do tônus adrenérgico, podem favorecer a ocorrência e a recorrência de arritmias cardíacas e da TE.

### 4.1. Antiarrítmicos

Os antiarrítmicos exercem seus efeitos sobre os canais iônicos das células do miocárdio, possibilitando, em determinadas situações, a reversão da arritmia ou facilitando a cardioversão elétrica, principalmente por meio da inibição de gatilhos que reinduzem as arritmias. Além disso, após a reversão, os medicamentos antiarrítmicos desempenham um papel essencial na prevenção da recorrência das arritmias ventriculares.^1,2,49^

De acordo com o mecanismo de ação, os medicamentos antiarrítmicos são categorizados segundo a classificação de Vaugham-Williams^50^, descrita na Tabela 1. As medicações disponíveis no Brasil também estão listadas na Tabela 1.^4^

**Tabela 1:** Classificação de Vaugham-Williams

Classe	Ação farmacodinâmica	Medicações disponíveis no Brasil
IA	Bloqueio moderado das correntes de Na+ e de K+ Velocidade intermediária de ligação e dissociação Podem aumentar o QRS e o QTc	—
IB	Reduzem excitabilidade elétrica Bloqueio leve das correntes rápidas e tardias de Na+ Velocidade rápida de ligação e dissociação Não aumentam o QTc	Lidocaína Fenitoína
IC	Bloqueio intenso das correntes rápidas e tardias de Na+ e leve da corrente de Ca++ e dos receptores beta Velocidade lenta de ligação e dissociação Aumentam o QRS	Propafenona
II	Betabloqueadores	Propranolol Succinato e tartarato de metoprolol Atenolol
III	Bloqueio de canais repolarizantes de K+ Aumentam o período refratário, dificultando a reentrada Aumentam o intervalo QT	Amiodarona Sotalol
IV	Bloqueadores de canais de Ca++	Verapamil Diltiazem

Os antiarrítmicos mais utilizados no atendimento emergencial de TE são a amiodarona e a lidocaína, devido à disponibilidade de infusão endovenosa.

### 4.2. Amiodarona

A amiodarona endovenosa é o medicamento de primeira escolha para o tratamento de TE.^51^ É também o antiarrítmico mais eficaz para o controle de arritmias ventriculares em pacientes com disfunção ventricular e/ou doença estrutural cardíaca, apresentando, em estudo clássico, uma taxa de reversão de aproximadamente 60%.^52^

Tem início de ação lento e, portanto, requer a administração de uma dose de ataque em velocidade de infusão mais rápida, seguida de infusão contínua, favorecendo a impregnação necessária para a eficácia esperada. Usualmente, utiliza-se uma dose inicial endovenosa de ataque de 6 a 7 mg/kg (em torno de 300 mg), com velocidade de infusão variando de 20 a 60 min, de acordo com a ausência ou presença de disfunção ventricular, respectivamente. A dose de manutenção varia de 900 a 1.200 mg/dia, mantida por 24 ou 48 h, em bomba de infusão contínua. Em casos selecionados, a dose máxima diária pode chegar a 2,2 g.

O uso endovenoso pode frequentemente induzir hipotensão, dependendo da dose e da velocidade de infusão, devendo-se atentar para seu uso em pacientes com sinais de baixo débito cardíaco ou instabilidade hemodinâmica.^21,22^ Após esse período, a medicação deve ser modificada para a via oral, sendo mantida na dose de 600 mg/dia até atingir uma dose cumulativa de 10 g, considerada adequada para impregnação.

A amiodarona tem efeitos colaterais que não devem ser desprezados. Possui a capacidade de prolongar o intervalo QT e, consequentemente, induzir efeitos pró-arritmogênicos. Por isso, é contraindicada em pacientes com TE associada a QT longo ou TVP. Durante o período de impregnação, deve-se considerar a realização diária de ECG de 12 derivações para acompanhamento do intervalo QT. Na presença de prolongamento do intervalo QT acima de 500 ms, sugere-se a redução da dose utilizada ou, até mesmo, a suspensão do medicamento.

O uso crônico da amiodarona está relacionado a depósitos corneais e fotossensibilidade (25-75% dos pacientes), aumento das enzimas hepáticas (30%), hepatite, cirrose (<3%), fibrose pulmonar e distúrbios tiroidianos.^53^

### 4.3. Lidocaína

Quando a amiodarona não é eficaz ou há alguma contraindicação ao seu uso, a lidocaína é considerada o medicamento de segunda linha, seja em associação à amiodarona ou em monoterapia. Apesar de sua eficácia ser menor à da amiodarona, a lidocaína é segura e relativamente mais bem tolerada em pacientes com isquemia miocárdica. Pode também ser associada a outras medicações que prolongam o intervalo QT.

Os efeitos adversos mais comuns são de natureza neurológica, incluindo agitação, ansiedade, confusão, letargia, perda da consciência, parestesia, psicose, convulsões e fala arrastada.^54^ Outros efeitos adversos, menos comuns, são redução de sensibilidade da via aérea por depressão dos reflexos e diminuição do influxo de cálcio no músculo liso.^55^

Utiliza-se uma dose de ataque de 1 a 1,5 mg/kg, administrada ao longo de aproximadamente 30 min, seguida de dose de manutenção de 1 a 4 mg/min.^4^

### 4.4. Betabloqueadores Adrenérgicos

A participação da estimulação adrenérgica na fisiopatologia da TE é bem estabelecida. Dessa forma, adicionar um betabloqueador adrenérgico aumenta a eficácia das medicações antiarrítmicas, reduzindo a recorrência da arritmia ventricular. Deve-se lembrar que a maior limitação ao uso desses fármacos ocorre em pacientes com sinais de baixo débito, congestão refratária ou em uso de medicamentos vasoativos na apresentação clínica da TE, uma vez que o uso de betabloqueadores pode favorecer ou precipitar choque cardiogênico.^48^

O uso de esmolol por via endovenosa contínua pode ser considerado devido à sua rápida ação, possibilidade de titulação e meia-vida curta, demonstrado resultados favoráveis.^34^ Entretanto, deve-se preferir o uso de betabloqueadores por via oral. A adição ou otimização da dose habitual dos betabloqueadores deve ser considerada em todo cenário de TE. Atenção maior deve ser dada à dose e à tolerância ao carvedilol, uma vez que esse fármaco tem também ação hipotensora pelo efeito bloqueador alfa 1 adrenérgico associado.^29^

Além dos betabloqueadores habitualmente utilizados em pacientes com arritmia ventricular associada à disfunção ventricular, como carvedilol e metoprolol, o uso de propranolol vem apresentando resultados interessantes. O propranolol tem efeito beta-1 e beta-2 adrenérgico não seletivo, apresentando também certo efeito no sistema nervoso central devido à sua natureza lipofílica. Assim, além do efeito antiarrítmico, favorece ação ansiolítica e age também em canais de sódio, potássio e cálcio nas células cardíacas, diminuindo a dispersão do intervalo QT.^14,15^

Um estudo clínico randomizado comparou o uso do propranolol na dose de 40 mg a cada 6 h com metoprolol 50 mg a cada 6 h, simultaneamente à infusão de amiodarona em pacientes portadores de CDI com TE. Foi observada uma redução no número de eventos de TV (razão de taxa de incidência: 0,375) e terapia do CDI (razão de taxa de incidência: 0,428) nos pacientes que receberam propranolol. Ao final das primeiras 24 h de tratamento, 90% dos pacientes do grupo propranolol não apresentavam mais TE, em comparação com 53,3% no grupo metoprolol. Outro ponto importante é que, a despeito dos efeitos inotrópicos negativos do propranolol, a medicação foi bem tolerada.^35^

### 4.5. Sulfato de Magnésio

O magnésio é o segundo íon mais prevalente no espaço intracelular e no miocárdio. Ele regula a movimentação pelos canais de sódio, potássio e cálcio durante a atividade elétrica e o acoplamento excitação-contração. O sulfato de magnésio, administrado por infusão endovenosa, tem ação antiarrítmica, sendo usualmente utilizada uma dose de 2-3 g.

Seu uso em pacientes com TE é indiscutível no cenário de TVP e prolongamento do intervalo QT, mesmo quando os valores séricos forem normais.^1,2,21,22,43^

Apesar de bem estabelecido nesse cenário, há poucas evidências que demonstram sua eficácia em pacientes com TVM, não sendo indicado para reversão de arritmia, mas sim como terapia adjuvante.

Aproximadamente 38% dos pacientes com TE apresentam deficiência de magnésio sérico e 72% apresentam fatores de risco que aumentam sua perda, como, por exemplo, o uso de diuréticos em altas doses. A correção e a manutenção dos níveis séricos de magnésio acima de 2 mg/dL reduzem a densidade de extrassístoles e os episódios de TV não sustentada, reduzindo a recorrência de TE.^56^

A Tabela 2 apresenta as recomendações de tratamento medicamentoso sugeridas na abordagem da TE.

**Tabela 2:** Recomendações de tratamento medicamentoso sugeridas na abordagem da tempestade elétrica

Medicações	Drogas antiarrítmicas	Bloqueadores adrenérgicos	Coadjuvantes
Primeira linha	Amiodarona IV: Bolus 300 mg (em torno de 6-7 mg/kg) em 20-60 min Manutenção: 900-1200 mg/dia por 24-48 h Repetir 150 mg se arritmia ventricular recorrente	Betabloqueadores orais: Propranolol 40 mg a cada 6 h; (preferencialmente) Metoprolol 25-50 mg a cada 12 h (com perspectiva de aumento da dose) Otimização das doses de carvedilol e bisoprolol usadas cronicamente	Sulfato de magnésio IV: 2-3 g em 30 min (obrigatório em pacientes com taquicardia polimórfica) Reposição para manter níveis adequados de magnésio sérico (> 2 mEq/L)
Segunda linha	Lidocaína IV: Bolus 1-1,5 mg/kg Manutenção: 1-4 mg/min.	Betabloqueadores IV: Esmolol: bolus 0,5 mg/kg seguido de infusão contínua de 50-300 mcg/kg/min.	Cloreto de potássio IV ou VO: Reposição para manter níveis adequados de potássio sérico (> 4 mEq/L).

IV: intravenoso; VO: via oral.

## 5. Manejo e Programação do Cardioversor-Desfibrilador Implantável

Pacientes com CDI podem evoluir com TE em 4 a 7% dos casos de prevenção primária e em 10 a 28% dos casos de prevenção secundária.^4,57^ A terapia elétrica com cardioversão, o desgaste muscular provocado pelas arritmias recorrentes e a ocorrência de apoptose e desequilíbrio autonômico e eletrolítico contribuem para um aumento de 2 a 3 vezes na mortalidade dos portadores de CDI com TE.^58^ Além disso, pacientes que recebem alta após a estabilização de um episódio de TE apresentam aumento semelhante na incidência de descompensação da insuficiência cardíaca nas semanas subsequentes.^14^ Ajustar adequadamente a programação do CDI, com o objetivo de otimizar as terapias, mantendo a eficácia e evitando prejuízos hemodinâmicos, é fundamental no manejo desses pacientes.^59^

### 5.1. Terapias do Cardioversor-Desfibrilador Implantável

**Figura 3:**
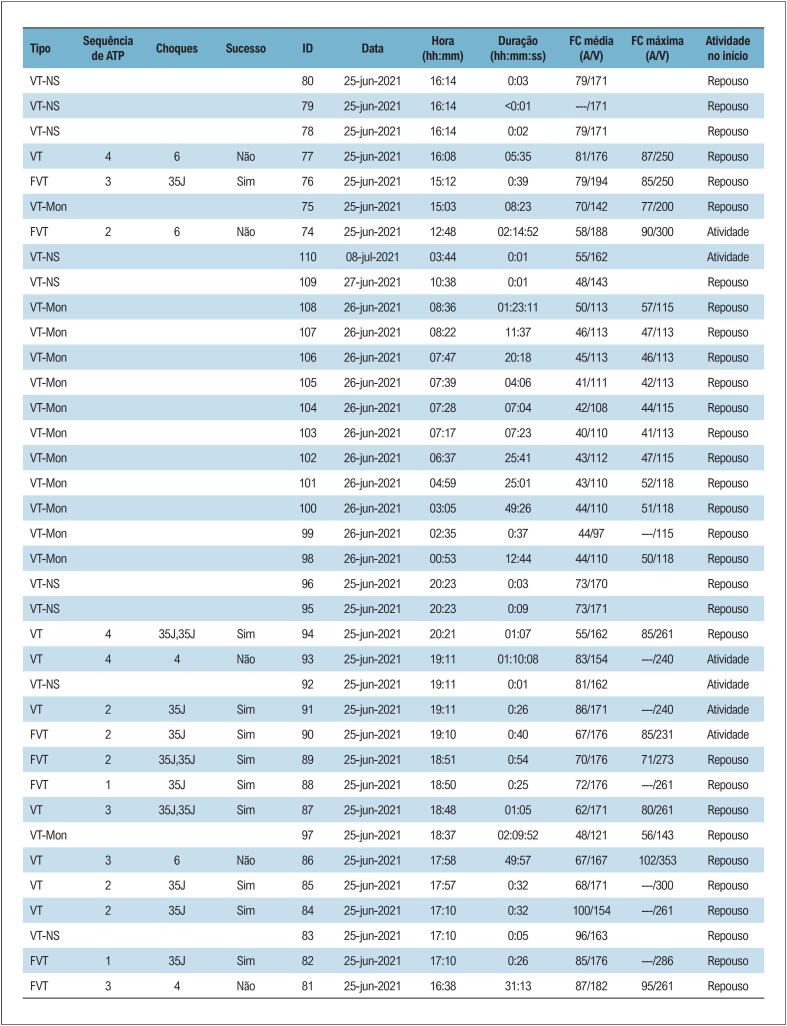
Registro de episódio de TE. Paciente recebeu 35 choques em um intervalo de 8 horas. Vários choques ocorreram em sequência, sem sucesso na reversão. Alguns episódios de TV ocorrem com frequência em torno de 110 (zona de monitorização, TV monomórfica) ou revertem espontaneamente (TV não sustentada). TE: tempestade elétrica; TV: taquicardia ventricular.

As terapias automáticas disponíveis nos CDIs atuais abrangem choques de alta ou baixa energia, estimulação artificial com frequência superior à da taquicardia (ATP) e a própria estimulação convencional, como aquela disponibilizada pelos marcapassos, necessária para suporte terapêutico naqueles pacientes que evoluem com bradicardia em uso de antiarrítmicos e betabloqueadores. A terapia de choque em pacientes com TE pode acontecer em duas situações diferentes: (1) terapia adequada em pacientes com arritmia ventricular sustentada potencialmente fatal ou (2) terapia precoce em pacientes com arritmia passível de resolução espontânea, como uma TV não sustentada.^60^ A aplicação de choques repetitivos em pacientes acordados, além de ser muito desconfortável, pode contribuir para a piora da função ventricular e ativar ainda mais o sistema adrenérgico, perpetuando a TE (Figura 3).^61^

### 5.2. Detecção de Eventos

As terapias entregues automaticamente pelo CDI podem ser escalonadas de acordo com a frequência cardíaca, classificada em diferentes zonas, e, conforme a programação escolhida, podem-se selecionar estratégias de intervenção mais ou menos agressivas. Nas respectivas zonas de detecção, define-se a TV, em uma ou duas zonas de frequências diferentes, a FV e a zona de monitorização, na qual o dispositivo pode armazenar os traçados de episódios de frequência elevada, sem a aplicação de terapias de choque ou ATP. Os algoritmos utilizados nos CDI detectam arritmias com base na frequência cardíaca, associada à verificação de critérios discriminatórios destinados a excluir arritmias supraventriculares. Quanto mais baixa for a frequência de detecção inicial com terapia programada, mais agressiva é a intervenção, com possibilidade de choques inclusive em situações de taquicardia sinusal. Por outro lado, programações que utilizam apenas frequências muito elevadas como critério de terapia em zona de FV (*shock box*) podem evitar terapias inapropriadas, mas inviabilizar a intervenção automática em episódios de TV com frequências mais baixas.

Outro aspecto importante no que se refere à detecção é o número de batimentos necessário para completar a contagem. O CDI considera uma taquiarritmia como detectada quando um número predefinido de batimentos em determinada frequência é reconhecido. Por exemplo, se o algoritmo reconhece uma zona de TV como sendo 16 batimentos com frequência de 160 bpm, a arritmia só será detectada a partir do 16º batimento consecutivo com frequência igual ou superior a 160 bpm. A maneira como essa contagem é feita interfere diretamente no intervalo entre o começo da arritmia e a liberação do choque ou da ATP. Existem outras formas de especificar esse atraso, dependendo da zona de detecção, como a utilização de um intervalo de tempo em segundos ou de um número mínimo de batimentos contados a partir de uma amostra (X em Y). Esses mecanismos são úteis na discriminação de arritmias não sustentadas ou de extrassístoles frequentes. Choques inapropriados podem ser evitados com o aumento do tempo de detecção; entretanto, o prolongamento exagerado desse intervalo pode comprometer a segurança do paciente diante de arritmias instáveis.^60^

### 5.3. Choque

**Figura 4:**
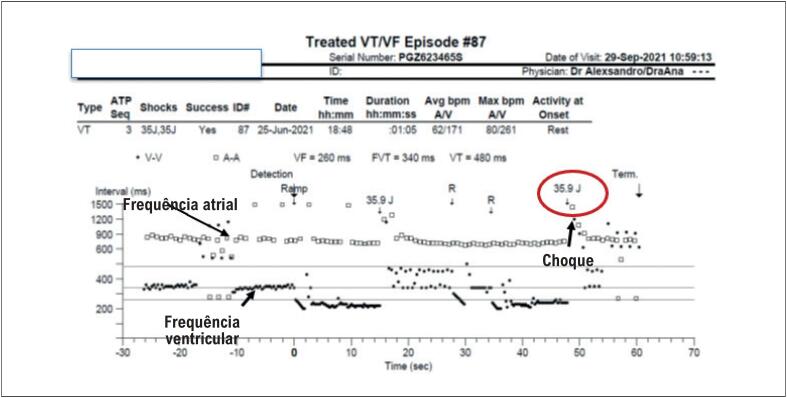
Gráfico de intervalos durante episódio de taquicardia ventricular. A frequência atrial está dissociada da ventricular. Observa-se uma tentativa de reversão com terapia antitaquicardia (ramp) e um choque inefetivo (35,9 J) antes do choque efetivo, que resulta no término da taquicardia.

A terapia mais característica do CDI é o choque. Liberado a partir da configuração de onda bifásica, a energia de desfibrilação é entregue ao coração através de eletrodos dotados de molas de choque, localizados no endocárdio ou, no caso dos CDIs subcutâneos, no tecido subcutâneo.^62^ A eficácia do choque em reverter uma determinada TV/FV depende do limiar de desfibrilação.^63,64^ É possível programar choques de menor energia para a reversão de TV monomórfica em zonas abaixo da zona de FV. Contudo, em casos de arritmias detectadas em zona de FV, embora seja possível tentar um choque inicial de 15 a 25 J, o escalonamento da terapia geralmente envolve uma sequência de choques sucessivos e progressivos até a energia máxima, em geral com até seis tentativas (Figuras 4 e 5).^65^

**Figura 5:**
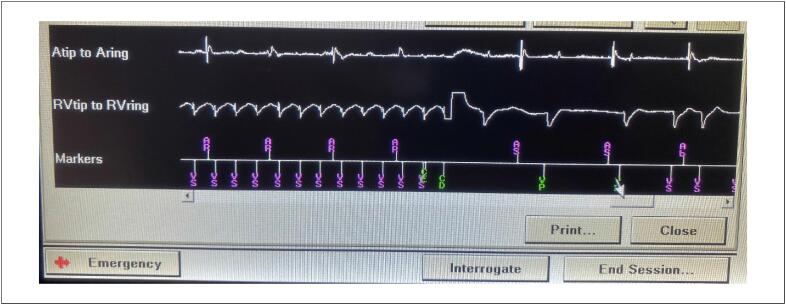
Traçado de registro intracavitário de uma taquicardia ventricular (parte esquerda da imagem), mostrando o eletrograma atrial (Atip to Aring) e o ventricular (RVtip to RVring). O choque apropriado (CD) é efetivo em restaurar o ritmo.

### 5.4. Terapia Antitaquicardia

**Figura 6:**
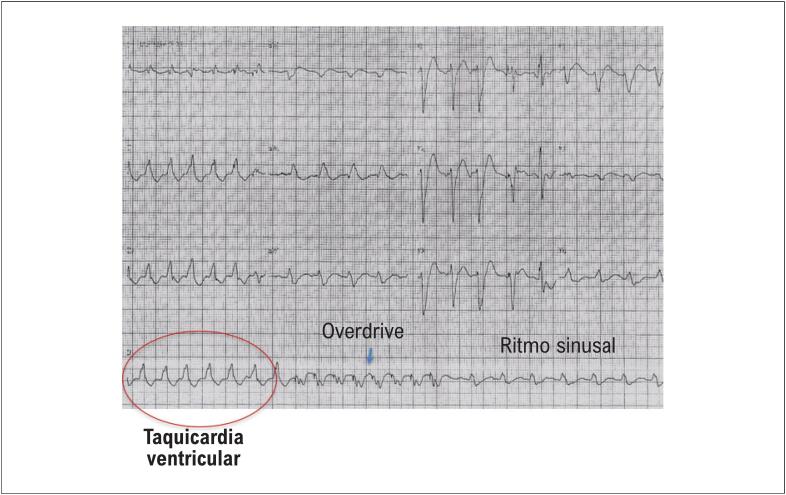
Exemplo de terapia antitaquicardia em taquicardia ventricular. Observa-se a terapia com oito pulsos com frequência acima da taquicardia (overdrive). O retorno ao ritmo sinusal ocorre sem necessidade de choque.

Os dispositivos de CDI inseridos por via transvenosa, à semelhança dos marcapassos, dispõem de eletrodos localizados na câmara ventricular, capazes de fornecer estimulação ventricular artificial. Nesse contexto, é possível reverter arritmias ventriculares reentrantes através de pulsos de estimulação ligeiramente acima da frequência da TV (Figura 6). O princípio dessa terapia envolve a quebra do circuito reentrante da taquicardia. A vantagem da programação da terapia com ATP é a reversão do episódio de TV sem o desconforto e os efeitos hemodinâmicos deletérios do choque.^65^ O estudo PainFREE avaliou a utilidade da programação de ATP em taquicardias com frequência de até 250 bpm, demonstrando reversão sem choque em 72% dos pacientes, com consequente redução de choques e melhoria da qualidade de vida.^66^

### 5.5. Estimulação Cardíaca Artificial

Todo CDI é um marcapasso com disponibilidade de cardioversão elétrica. A estimulação convencional para o controle de bradiarritmia pode ser necessária em pacientes com arritmia ventricular e TE. A configuração do CDI pode ser unicameral ou bicameral, podendo inclusive estar associada à terapia de ressincronização. Dessa forma, qualquer portador de CDI pode receber diferentes modos de estimulação artificial baseados em átrio ou ventrículo (por exemplo, AAI e DDD). Pacientes que evoluem com TE recebem terapias agressivas, que incluem medidas farmacológicas ou não, capazes de exacerbar a bradicardia. Assim, é preciso atentar para a programação antibradicardia, a fim de garantir uma frequência cardíaca fisiológica adequada à condição do paciente. Muitas vezes, a incompetência cronotrópica ou a presença concomitante de um defeito de condução atrioventricular pode comprometer a estabilização hemodinâmica do paciente com TE.^67^

### 5.6. Abordagem do Paciente com Cardioversor-Desfibrilador Implantável em Tempestade Elétrica

Os pacientes que evoluem com TE na presença de CDI frequentemente recebem múltiplos choques. Além de aumentar o estresse e a ansiedade e ser extremamente doloroso, o choque repetitivo eleva a atividade adrenérgica, o que contribui para perpetuar a deflagração de arritmias ventriculares e aumentar a mortalidade. Às vezes, os pacientes chegam à sala de emergência conscientes, com pressão arterial estável e recebendo choques na presença da equipe médica. A colocação de um ímã sobre a loja do CDI desabilita as terapias de choque enquanto o dispositivo estiver sob a abrangência do campo magnético. Essa intervenção pode ser útil para acalmar o paciente e aliviar a dor, permitindo que, sob monitorização, ele receba sedação adequada, antiarrítmicos venosos, suporte de oxigênio e estabilização do quadro, evitando a ocorrência de choques enquanto estiver acordado.

Após a abordagem inicial, é importante providenciar imediatamente a interrogação do dispositivo. Por meio de telemetria, utilizando o equipamento apropriado de programação para o respectivo CDI, é possível avaliar a causa dos choques (inclusive determinar se foram inapropriados ou não) e entender os mecanismos deflagradores da TE. Arritmias polimórficas, TV monomórfica e FV podem ter formas diferentes de iniciação, que podem ocorrer após extrassístoles de acoplamento curto, após pausas ou após TVNS. É preciso considerar, nesse momento, mudanças na forma de detecção, aumento na frequência básica de estimulação e, até mesmo, a desabilitação temporária das terapias com choque.^68^

Eventualmente, a estimulação epicárdica do ventrículo esquerdo, forma clássica de terapia de ressincronização, pode deflagrar TE por dispersão heterogênea do potencial de ação ou mesmo pela indução de TV reentrante em circuitos muito estáveis. Nesses casos de TE induzida pela estimulação do ventrículo esquerdo em terapia de ressincronização com CDI biventricular, já foram descritas situações em que a estabilização foi alcançada com a desabilitação da estimulação do VE.^69^

Para pacientes com TV monomórfica reentrante e para aqueles com episódios frequentes de TV não sustentada, a programação de tempos de detecção prolongados e o uso de terapias com ATP são eficazes na redução do número de choques.^70^ Mesmo em pacientes com TE com grande número de arritmias, ocorrem episódios significativos de TV autolimitada que não necessitam de intervenção do CDI. A adoção de critérios de detecção cuidadosamente ajustados, com tempo suficiente para evitar choques em TV não sustentada, melhora o conforto do paciente durante a TE. As terapias com ATP são capazes de reverter boa parte dos episódios em zona de TV, inclusive em alguns casos em que a taquicardia se apresenta muito rápida em zona de FV nos momentos iniciais.

**Figura 7:**
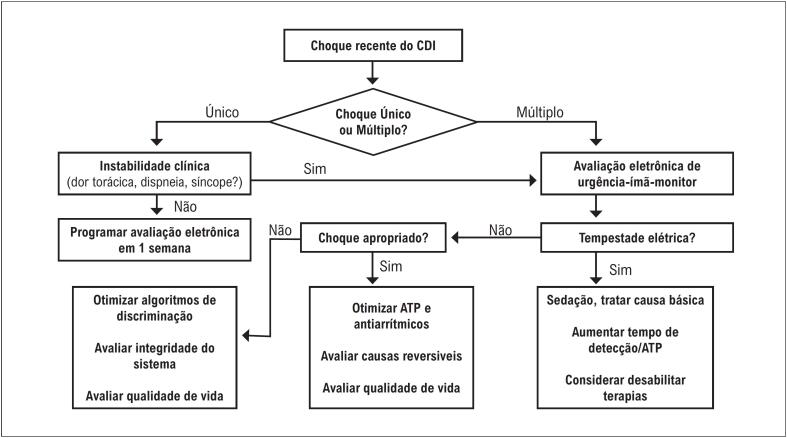
Fluxograma de abordagem e programação emergencial em pacientes com terapias aplicadas pelo CDI. ATP: terapia antitaquicardia; CDI: cardioversor-desfibrilador implantável.

A abordagem da programação do CDI na TE deve priorizar a segurança do paciente e a minimização dos danos. Aumentar o tempo de detecção, ajustar a frequência e o modo de estimulação de acordo com o contexto de cada paciente e privilegiar, sempre que possível, a reversão da TV com ATP são ações que devem nortear os princípios de programação. Eventualmente, a desabilitação imediata com a colocação de um ímã sobre a loja do CDI e/ou a reprogramação para desabilitar as terapias pode ser necessária em casos de choques recorrentes, desde que a equipe disponha de materiais de suporte e monitorização adequados. A Figura 7 apresenta um fluxograma de abordagem do CDI no paciente com TE.

## 6. Ablação por Cateter no Contexto de Tempestade Elétrica

A ablação por cateter da TV é um procedimento de extrema importância em pacientes com TE, principalmente quando a TV é monomórfica. Após a abordagem clínica inicial, pacientes que apresentam quadros recorrentes de TV têm indicação de ablação por cateter em caráter de emergência.^1,2,43,71-73^ Embora sejam procedimentos mais complexos, a estratégia de ablação é semelhante à utilizada em outras situações de TV, envolvendo o mapeamento da arritmia e a modificação do substrato com base em mapeamentos funcionais. Pacientes com TE associada à síndrome de Brugada^74^ e à FV^75,76^ também podem ser submetidos à ablação, porém com técnicas específicas para essas situações.

Carbucicchio et al.^77^ investigaram prospectivamente 95 pacientes com cardiopatia isquêmica e não isquêmica que foram submetidos a ablação por cateter em um contexto de TE refratária a medicamentos antiarrítmicos. Em 62 pacientes, nenhuma TV foi mais induzível (sucesso completo); em 19, a TV clínica não foi mais induzível (sucesso parcial), mantendo-se a indução de TV não clínica; e, em 14, a TV clínica ainda se mantinha induzível (insucesso). Em 12 pacientes, foi necessário um segundo procedimento e, em seis, um terceiro. Entre os pacientes com sucesso completo e sucesso parcial, nenhum apresentou recorrência da TE. Já entre os pacientes com insucesso após o último procedimento, oito de 10 (80%) apresentaram recorrência da TE. Em um seguimento de 22 meses, 15 (16%) pacientes morreram, 11 (12%) por causas cardíacas, quatro deles por morte súbita, sendo que, em todos esses pacientes, o resultado da ablação havia sido insucesso. Os pacientes com sucesso parcial apresentaram, na maioria das vezes, recorrência de TV no seguimento (65%), porém não houve recorrência de TE. Já os pacientes com sucesso completo (16%) apresentaram recorrência de TV. Não se observou morte relacionada ao procedimento.

Mais recentemente, Muser et al.^78^ também investigaram pacientes com miocardiopatia isquêmica e não isquêmica com TE (n=267). O objetivo do procedimento era eliminar a TV clínica e, se possível, outras taquicardias ventriculares mapeáveis. Em um seguimento mediano de 45 meses, 76 (29%) dos pacientes morreram e 25 (9%) foram submetidos a transplante cardíaco. Oitenta e sete (33%) pacientes apresentaram recorrência de TV, porém apenas 13 (5%) apresentaram recorrência da TE.

Nos pacientes com TE associada a fibrilação ventricular, mesmo em fase aguda de infarto do miocárdio, a ablação por cateter pode ser uma opção. O principal alvo nesses casos é a extrassístole que deflagra a FV. A ablação também só deve ser indicada em casos de refratariedade ao tratamento medicamentoso, já otimizados do ponto de vista isquêmico e com suporte hemodinâmico também otimizado. O estudo de Komatsu et al.^76^ incluiu 110 pacientes que apresentaram TE devido a FV após infarto do miocárdio. A maioria dos casos ocorreu na fase aguda (39%) e subaguda (44%) do infarto, ou seja, nos primeiros meses após o infarto. A TE foi controlada em 92 pacientes (84%), e 30 (27%) apresentaram mortalidade intra-hospitalar. Apenas um paciente que recebeu alta apresentou recorrência de TE em um seguimento médio de 2,2 anos. Entretanto, 29 pacientes (36%) faleceram nesse período de seguimento.

Uma ferramenta que pode ser utilizada para estimar o risco de recorrência de TE após ablação é o escore PAINESD. Pacientes com escore alto (> 15 pontos) podem se beneficiar de suporte hemodinâmico periprocedimento. O escore considera:^1,2^

P – Doença pulmonar crônica – 5 pontos;

A – Idade (*age*) > 60 anos – 3 pontos;

I – Infarto agudo do miocárdio prévio ou cardiomiopatia isquêmica – 6 pontos;

N – Classe funcional NYHA > III – 6 pontos;

E – Fração de ejeção < 25% - 3 pontos;

S – Choque cardiogênico prévio – 9 pontos;

D – Diabetes *mellitus* – 3 pontos.

### 6.1. Terapias Adicionais para Tempestade Elétrica

A hiperatividade simpática exerce um papel fundamental no desenvolvimento e na manutenção das arritmias. O aumento da atividade adrenérgica aumenta a automaticidade, reduz o período refratário efetivo ventricular e reduz o limiar para a ocorrência de arritmias. Logo, a modulação do eixo cardioneural pode ser uma alternativa no tratamento das arritmias ventriculares malignas, especialmente a TE.^29^

A eficácia do bloqueio simpático no controle da TE foi demonstrada há mais de 20 anos, com efeitos favoráveis dos betabloqueadores endovenosos na redução de episódios de FV e no aumento da sobrevida em curto prazo.

Além dos betabloqueadores, a modulação do eixo cardioneural pode ser obtida por meio de técnicas como sedação, anestesia torácico-epidural, bloqueio do gânglio estrelado, denervação simpática cardíaca cirúrgica e denervação simpática renal.

#### 6.1.1. Sedação

A sedação desempenha um papel fundamental no arsenal terapêutico da TE. Os principais objetivos da sedação nesse contexto são reduzir os efeitos pró-arrítmicos do tônus simpático, aliviar o estresse psicológico e alcançar estabilização hemodinâmica.

A sedação deve ser adaptada aos diferentes cenários clínicos, podendo variar desde superficial, para alívio da dor e sofrimento psicológico, até sedação profunda, com intubação orotraqueal e ventilação mecânica.

A sedação profunda pode ser considerada nos casos de ausência de resposta ao tratamento com antiarrítmicos e ocorrência de múltiplos choques em curto intervalo de tempo. Nesse contexto, a hiperatividade simpática após terapias repetidas do CDI resulta em agravamento clínico e no desencadeamento de novas arritmias ventriculares, contribuindo para um ciclo vicioso.^79^

Os medicamentos mais comumente prescritos são os benzodiazepínicos (por exemplo, midazolam), dexmedetomidina e os analgésicos de curta duração (por exemplo, fentanil), que promovem ação eficiente sem efeitos inotrópicos negativos.

O propofol possui diversas vantagens que justificam seu uso em ambiente cardiovascular, como início rápido de ação (2 a 3 minutos), meia-vida curta e potencial amnésico. No entanto, sua estreita janela terapêutica exige monitorização hemodinâmica cuidadosa. Geralmente, o propofol é administrado em *bolus* seguido por infusão contínua. Seus principais efeitos colaterais incluem bradicardia, hipotensão e depressão respiratória. Embora raro, choque cardiogênico pode ocorrer, dado o efeito inotrópico negativo do medicamento.^80^

Além de reduzir a atividade simpática e aumentar o tônus vagal, o propofol modifica a sensibilidade barorreflexa e a condução atrioventricular. Adicionalmente, o propofol atua diretamente no cardiomiócito por meio de alterações na translocação da proteína quinase C para diferentes alvos intracelulares.

Um estudo com 15 pacientes com TE refratária ao tratamento inicial, submetidos a sedação profunda (primariamente com propofol e infusão concomitante ou subsequente de outros agentes, incluindo fentanil) com intubação orotraqueal, demonstrou resolução completa da taquicardia ventricular em 80% dos casos e parcial em 13%.^81^

Em estudo retrospectivo multicêntrico conduzido por Martins et al., a eficácia da sedação profunda foi avaliada em 116 pacientes com TE refratária aos antiarrítmicos. Os agentes hipnóticos e bloqueadores neuromusculares de ação rápida foram utilizados para indução e intubação, seguidos por administração de um hipnótico intravenoso (midazolam ou propofol) e opioides para manutenção. A resposta aguda, observada em 15 minutos, ocorreu em quase metade dos casos e constituiu preditor independente de melhor sobrevida intra-hospitalar, associando-se a um risco 55% menor de óbito.^82^

A dexmedetomidina se destaca pelo menor risco de depressão respiratória, além de efeito ansiolítico e supressão simpática. Estudos de caso sugerem que ela pode reduzir a frequência de choques do CDI e de recorrência de TV, podendo ser útil inclusive em pacientes instáveis.^2^

#### 6.1.2. Anestesia Torácico-Epidural

A anestesia torácico-epidural consiste na infusão de um anestésico local de longa duração, como a bupivacaína ou a ropivacaína, no espaço peridural, entre as vértebras T1-T2 ou T2-T3. O procedimento pode ser realizado à beira leito ou com auxílio de fluoroscopia. Trata-se uma terapia efetiva, de efeito rápido e curta duração, que pode ser considerada em casos de taquicardia ventricular incessante refratária. No entanto, essa técnica é contraindicada em pacientes que necessitam de dupla antiagregação ou anticoagulação.^83^ O mecanismo antiarrítmico proposto é a redução do tono simpático cardíaco, com consequente prolongamento do potencial de ação e do período refratário.^84^

Dados da literatura nesse tópico são escassos. Bourke et al. demonstraram redução de 80% na carga de arritmia ventricular em 75% dos pacientes não responsivos à ablação submetidos à anestesia torácico-epidural.^85^ Em outra publicação, 45% dos pacientes apresentaram respostas favoráveis.^41^

Do ponto de vista prático, o perfil de segurança favorável e as baixas taxas de hipotensão tornam a anestesia torácico-epidural uma alternativa promissora à sedação profunda com intubação orotraqueal prolongada em pacientes não responsivos a antiarrítmicos e/ou à ablação.^41^

#### 6.1.3. Bloqueio do Gânglio Estrelado

O bloqueio do gânglio estrelado é um procedimento realizado à beira do leito, guiado por fluoroscopia ou, preferencialmente, por ultrassom. Envolve a injeção local de anestésico (bupivacaína ou ropivacaína) no gânglio estrelado esquerdo, com o objetivo de reduzir temporariamente a atividade adrenérgica por meio do bloqueio de vias nervosas aferentes e eferentes. Pode ser considerado uma alternativa em pacientes refratários à ablação percutânea ou um método temporário até que medidas definitivas de bloqueio adrenérgico sejam estabelecidas. O bloqueio bilateral deve ser reservado para pacientes intubados, em virtude do risco de paralisia do nervo frênico, com comprometimento respiratório. Um estudo demonstrou redução de 93% na ocorrência de arritmias ventriculares agudas e redução de 90% na densidade arrítmica em 56% dos pacientes, durante um seguimento superior a 3 anos, quando o bloqueio do gânglio esquerdo foi combinado com a denervação simpática cardíaca.^86^

Uma série,^87^ outro estudo randomizado^29^ e metanálises mostraram benefícios desse procedimento na supressão temporária de arritmias ventriculares^42,88^ com segurança e efetividade satisfatórias.

#### 6.1.4. Denervação Simpática Cardíaca Cirúrgica

A denervação simpática cardíaca cirúrgica promove bloqueio neuroaxial permanente das inervações aferentes e eferentes cardíacas. Em 2014, Vasegui et al. avaliaram os efeitos da denervação simpática cardíaca em 41 pacientes com arritmias ventriculares refratárias ou TE e demonstraram reduções significativas no número de choques pelo CDI a médio e longo prazo, tanto com a denervação unilateral quanto bilateral, com efeitos mais favoráveis com a denervação bilateral.^89^ Os efeitos colaterais tardios (> 3 meses após o procedimento) incluíram alterações de sensibilidade, sudorese e ptose.

O procedimento é usualmente realizado por videotoracoscopia e envolve a ressecção de parte do gânglio estrelado e do segundo ao quarto gânglio torácico. Os benefícios desse tipo de intervenção foram descritos em pacientes com síndrome do QT longo e TVP catecolaminérgica.^90^

#### 6.1.5. Denervação Simpática Renal

A denervação renal percutânea consiste em uma abordagem endovascular seletiva e minimamente invasiva, associada a baixas taxas de complicações, sem eventos adversos sistêmicos descritos. O procedimento envolve a ablação do plexo neural localizado na adventícia da artéria renal, promovendo redução do efluxo simpático central para o coração.

Diversos estudos demonstraram os benefícios da denervação renal no tratamento da TE, incluindo a redução do número de episódios de arritmia ventricular e de terapias apropriadas do CDI em pacientes com arritmias ventriculares refratárias ao tratamento otimizado com antiarrítmicos e ablação. O procedimento mostrou-se seguro, sem associação com eventos adversos significativos ou alterações na pressão arterial ou função renal.^91^ A experiência com denervação renal em pacientes chagásicos é limitada e advém de um único estudo, que demonstrou a factibilidade e a segurança desse procedimento na redução de terapias do CDI, podendo ser considerado em casos refratários ou com contraindicação à ablação por cateter.^92^

#### 6.1.6. Manejo Circulatório

Em pacientes instáveis, com comprometimento hemodinâmico e episódios recorrentes de TV/FV com síncope, o suporte mecânico com dispositivos de assistência ventricular esquerda ou a oxigenação por membrana extracorpórea (ECMO) pode ser considerado como ponte ou suporte para ablação por cateter ou transplante cardíaco.^93^

Entre os dispositivos disponíveis, destacam-se a ECMO, o suporte circulatório TandemHeart, a bomba de balão intra-aórtico (BIA) e o dispositivo Impella.

#### 6.1.7. Oxigenação por Membrana Extracorpórea

Além de garantir a perfusão tecidual e reduzir a pressão diastólica final do ventrículo esquerdo, a ECMO ventriculoarterial oferece benefícios adicionais, como a capacidade de atingir níveis adequados de antiarrítmicos e facilitar o desmame rápido de catecolaminas.^93^ Esses efeitos combinados contribuem para a restauração e a manutenção do ritmo sinusal de forma satisfatória. Le Pennec-Prigent et al. reportaram restauração do ritmo sinusal após 3 horas em 61,5% dos 26 pacientes com TE refratária e choque cardiogênico submetidos à ECMO.^94^

As complicações, os custos elevados e a alta taxa de mortalidade em pacientes com taquicardia ventricular refratária limitam o uso da ECMO ventriculoarterial a centros especializados.^95^

#### 6.1.8. Balão Intra-Aórtico

A eficácia do BIA no tratamento de arritmias refratárias em pacientes com doença coronariana é amplamente reconhecida. No entanto, os dados em não coronariopatas são escassos. Seu baixo custo, ampla disponibilidade, facilidade de uso e baixa invasividade tornam o BIA uma ferramenta a ser considerada em situações que requerem suporte ventricular esquerdo de baixo a moderado grau.^96^

#### 6.1.9. Suporte circulatório TandemHeart e Dispositivo Impella

Algumas vantagens foram demonstradas com o uso de dispositivos de assistência ventricular, como o Impella e o TandemHeart. O Impella promove a redução da pressão de enchimento e da pós-carga, resultando em melhora significativa na perfusão miocárdica e proporcionando efeitos cardioprotetores.^97^ O Impella, entretanto, apresenta limitações no fornecimento de suporte hemodinâmico adequado em casos de TVs instáveis.

#### 6.1.10. Escolha do Dispositivo

A escolha do dispositivo depende da gravidade, das características individuais do paciente e da disponibilidade dos recursos técnicos e humanos necessários para o manejo adequado. O uso profilático de dispositivos de assistência ventricular percutânea em pacientes com alto risco de comprometimento hemodinâmico durante a ablação de TE apresenta resultados melhores em comparação ao uso de resgate. Em um estudo conduzido por Musser et al., o uso preventivo desses dispositivos resultou em redução da descompensação hemodinâmica durante a ablação, além de menor taxa de mortalidade e necessidade de transplante cardíaco.^98^ No estudo de Mathuria et al., o uso de dispositivos de resgate durante a ablação foi associado a altas taxas de mortalidade em 30 dias.^99^

Uma revisão sistemática com 2.465 pacientes demonstrou redução nas taxas de mortalidade com o suporte circulatório mecânico profilático no tratamento de apoio em pacientes com TE e escore PAINESD de alto risco. Já suporte circulatório mecânico de resgate durante a ablação foi associado a altas taxas de mortalidade.^100^

## 7. Manejo da Tempestade Elétrica Associada a Canalopatias

Em pacientes sem doença cardíaca estrutural, a ocorrência de TE pode estar associada a canalopatias. As principais canalopatias são a síndrome de Brugada, a síndrome do QT longo ou curto e a TVP catecolaminérgica. Outras doenças possuem fisiopatologias menos conhecidas, porém exibem um comportamento semelhante às canalopatias, com potencial de causar TVP rápida, TdP e FV, mesmo na ausência de uma cardiopatia estrutural significativa. Nesse sentido, também se enquadram a síndrome de Haissaguerre (ou repolarização precoce maligna) e TdP desencadeado por extrassístoles ventriculares com acoplamento ultracurto (TdP-AUC), um tipo de TVP.

Em pacientes com diagnóstico pré-estabelecido, é possível ter um alvo terapêutico. No entanto, quando a TE ocorre como a primeira manifestação da doença, os fatores desencadeadores da arritmia (exercício, emoções, intoxicações, entre outros) podem auxiliar na estratégia terapêutica adicional às medidas básicas, como correção dos distúrbios eletrolíticos.

**Figura 8:**
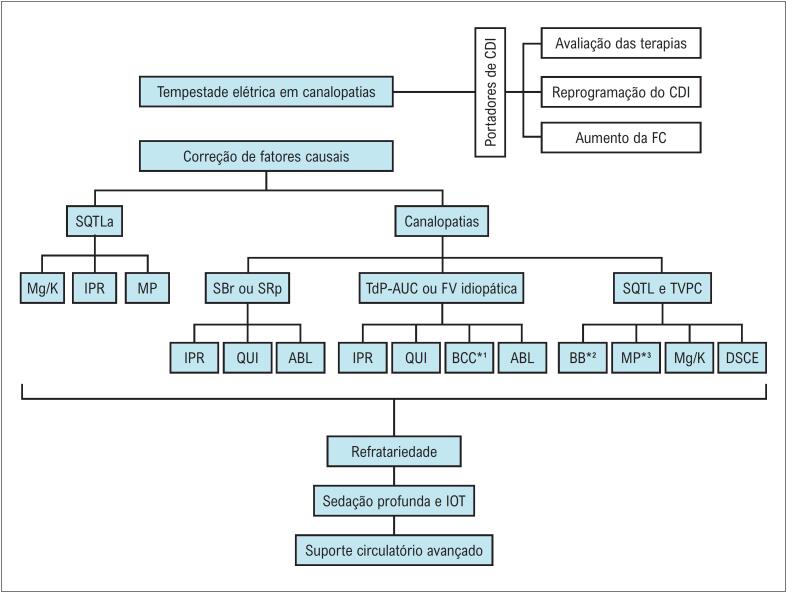
Manejo da tempestade elétrica em canalopatias. ABL: ablação por radiofrequência; BB*2: betabloqueadores endovenosos; BCC*1: bloqueadores dos canais de cálcio (verapamil); CDI: cardioversor-desfibrilador implantável; DSCE: denervação simpática cardíaca esquerda; FC: frequência cardíaca; IOT: intubação orotraqueal; IPR: isoproterenol; Mg/K: reposição de potássio e magnésio; MP*3 marcapasso programado com frequência cardíaca maior ou igual a 70 bpm; MP: marcapasso; QUI: quinidina; SBr: síndrome de Brugada; SQTL: síndrome do QT longo congênito; SQTLa: síndrome do QT longo adquirido; SRp: síndrome da repolarização precoce; TdP-AUC: *torsades de pointes* desencadeado por extrassístoles ventriculares de acoplamento ultracurto; TVPC: taquicardia ventricular polimórfica catecolaminérgica.

A exemplo do que ocorre nos casos de TE "*senso latu*", nos casos relacionados às canalopatias, também se deve levar em consideração as seguintes três principais premissas: 1) controle dos fatores deflagradores (distúrbios eletrolíticos, pró-arritmias etc.); 2) controle da arritmia *per se* (incluindo reprogramação específica do CDI, tratamento medicamentoso e/ou ablação por cateter); e 3) modulação autonômica (betabloqueadores, denervação simpática e sedação)^101^ (Figuras 8 e 9).

**Figura 9:**
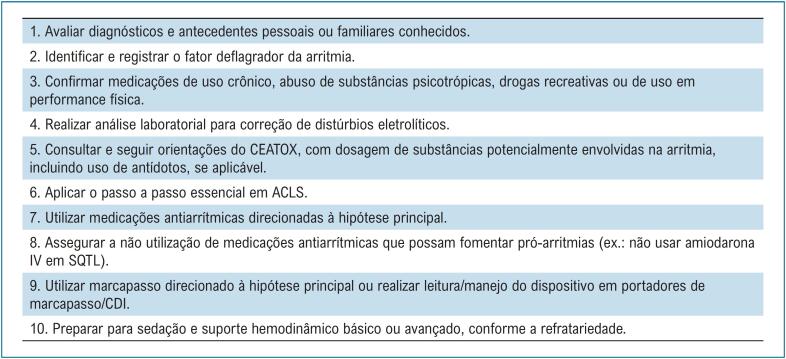
Os 10 recados mais importantes no manejo da tempestade elétrica em pacientes com canalopatias. ACLS: Advanced Cardiovascular Life Support (Suporte Avançado de Vida em Cardiologia); CEATOX: Centro de Informação e Assistência Toxicológica; CDI: cardiodesfibrilador implantável; IV: intravenoso; SQTL: síndrome do QT longo congênito.

O magnésio endovenoso é frequentemente usado para TE. É um modulador transmembrana e intracelular essencial da atividade elétrica das células cardíacas. Pode ser administrado tanto em paciente com níveis baixos (hipomagnesemia) quanto normais de magnésio no sangue. O magnésio costuma ser bem tolerado, mesmo em situações hemodinamicamente instáveis; além disso, sua aplicação é rápida e simples. O magnésio é o tratamento de escolha PARA pacientes com taquicardia tipo TdP, intervalo QT longo e arritmias cardíacas induzidas por digitálicos.^102^

O aumento do tônus simpático desempenha um papel fundamental no desenvolvimento e na manutenção de muitas arritmias ventriculares.^103^ Na TE, pode ocorrer um ciclo vicioso, principalmente em pacientes com CDI, em que os choques podem precipitar descarga simpática, resultando em mais arritmias ventriculares e choques, e assim por diante.^104^ Os betabloqueadores não seletivos, como o propranolol, são frequentemente usados nas canalopatias adrenérgico-dependentes, como TVP catecolaminérgica e SQTL. Em contraste, o início da arritmia em determinadas condições, como síndrome de Brugada, repolarização precoce, síndrome do QT curto e TdP-AUC, geralmente ocorre durante o sono ou em repouso – situações marcadas por aumento do tônus parassimpático. Nesses contextos, o uso de isoproterenol pode ser eficaz na prevenção de recorrências da arritmia.^105^

O manejo da TE inclui também orientações sobre condutas a serem evitadas. Para esse propósito, existem *sites* específicos para consulta sobre medicamentos contraindicados na síndrome de Brugada e na síndrome do QT longo, a saber: brugadadrugs.org e crediblemeds.org, respectivamente (Figura 9). Na síndrome do QT curto, considerando que o uso de lidocaína encurta o intervalo QTc, esse fármaco também deve ser evitado.

Nos pacientes com síndrome de Brugada, a arritmia incessante pode ser tratada com infusão endovenosa de isoprenalina,^106^ seguida por quinidina oral (ou cilostazol, se refratária à quinidina),^105^ porém a terapêutica com quinidina esbarra na falta de acesso em grande parte do mundo.^107^ O sotalol oral pode ser uma opção, desde que haja monitoramento do intervalo QTc e avaliação da resposta individual de cada paciente.^108^ A ablação por cateter do trato de saída ventricular direito pericárdico, visando à abordagem inicial de áreas de cicatriz de baixa voltagem e/ou áreas de condução lenta (ou provocadas por ajmalina intravenosa), mostrou-se altamente eficaz na síndrome de Brugada.^74^

Paralelamente, a presença da síndrome de repolarização precoce maligna, ainda sem fisiopatologia conhecida, porém semelhante à da síndrome de Brugada, direciona o tratamento da TE para o mesmo racional, com infusão de isoproterenol seguida de quinidina. Especificamente na síndrome de Brugada, múltiplos choques inapropriados podem ser desencadeados por *oversensing* da onda T e devem ser prontamente resolvidos pela reprogramação do CDI.^109^

O isoproterenol pode ser utilizado para aumentar a frequência cardíaca em pacientes sabidamente portadores de QT longo adquirido, como medida de preparo para a inserção do marcapasso. Seus efeitos incluem o aumento da frequência cardíaca por meio da ação agonista não seletiva sobre os receptores beta1/beta2-adrenérgicos, encurtando o intervalo QT e o período refratário efetivo quando os canais de potássio são normofuncionantes. Por outro lado, em pacientes com síndrome do QT longo congênito, em que os canais de potássio apresentam redução da função, o isoproterenol gera prolongamento do intervalo QT e indução de pós-potenciais precoces, aumentando a dispersão da repolarização e, consequentemente, gerando mais TE.^110^

Na SQTL congênita, além da administração intravenosa de magnésio e do uso de propranolol por via oral, quando possível, os pacientes podem necessitar de marcapasso provisório para manter uma frequência cardíaca elevada, suprimindo, assim, as extrassístoles. Adicionalmente, pode ser necessário utilizar lidocaína intravenosa, tanto em bolus quanto em infusão contínua, para manutenção. É muito importante, nos casos que exigem estimulação cardíaca do marcapasso, que a frequência de estimulação mínima fique acima de 80 bpm, evitando as arritmias malignas dependentes de pausas, como na SQTL do tipo 2 ou nas induzidas por fármacos.^111^

Na rara síndrome do QT curto, o manejo inicial pode ser realizado com isoproterenol endovenoso,^112^ sendo também possível o uso de quinidina,^113^ apesar de sua indisponibilidade no Brasil. Embora não existam grandes evidências, devido à raridade dos casos, há relatos de casos com potencial benefício desse medicamento em doses elevadas em situações específicas.^114^

Na TVP ou bidirecional, característica da TVP catecolaminérgica, os betabloqueadores são o tratamento de escolha, mas a sedação pode ser um complemento útil na urgência, assim como propranolol e propafenona orais. Se o tratamento for insuficiente ou não for tolerado para diminuir a atividade simpática, pacientes selecionados podem se beneficiar da modulação autonômica, ou seja, do bloqueio do gânglio estrelado percutâneo, da anestesia epidural-torácica ou da denervação simpática cardíaca esquerda.^115^

Alguns pacientes, independentemente do tipo de canalopatia, podem apresentar extrassístoles ventriculares, especialmente as originadas no sistema de Purkinje (banda moderadora do ventrículo direito, fascículo anterior ou posterior no ventrículo esquerdo), nos tratos de saída do ventrículo esquerdo, no anel tricúspide e nos músculos papilares, as quais desencadeiam a TE. Nesses casos, o alvo da ablação pode ser direcionado à extrassístole deflagradora. Uma variante peculiar de TE é o TdP-AUC, um tipo de TVP. Sua fisiopatologia ainda é desconhecida e não pode ser considerada uma canalopatia. Essa condição pode ser identificada na documentação da taquicardia do tipo TdP, mesmo na ausência de isquemia, doença cardíaca estrutural ou prolongamento do intervalo QT. O uso de verapamil em altas doses (em média até 480 mg/dia) também está indicado nessa rara situação de TdP-AUC.^116^

A Tabela 3 resume as principais recomendações de escolha de fármacos e uso do marcapasso em situações de emergência.

**Tabela 3:** Medicamentos endovenosos e MP no tratamento de tempestade elétrica em canalopatias

	Sim	Não
Magnésio	SQTL, TdP-AUC	**-**
Isoproterenol	SBr, SRp, SQTLa, SQTC	**SQTL**
Verapamil	TdP-AUC	
Lidocaína	SQTL	**SQTC?**
Esmolol	TVPC	
Amiodarona	SQTC?	**SQTL, SQTLa, SBr, TVPC**
MP	SQTL, SQTLa	

SQTL: síndrome do QT longo congênito; SQTLa: síndrome do QT longo adquirido; SQTC: síndrome do QT curto; SBr: síndrome de Brugada, SRp: síndrome da repolarização precoce maligna; TVPC: taquicardia ventricular polimórfica catecolaminérgica; TdP-AUC: **torsades de pointes** desencadeado por extrassístoles de acoplamento ultracurto; MP: marcapasso provisório com frequência de estimulação acima de 80 bpm.

Os fármacos orais de manutenção, específicos para cada canalopatia, devem ser introduzidos ou reintroduzidos precocemente, como mexiletina na SQTL tipo 3, nadolol ou propranolol na SQTL tipos 1 e 2, sotalol na síndrome de Brugada (na ausência de quinidina) ou na SQTC e propafenona na TVP catecolaminérgica.^101^

## 8. Prevenção da Recorrência a Longo Prazo

A ocorrência de TE está associada a aumento da mortalidade tanto na fase aguda como a longo prazo.^2^ Assim, após a estabilização inicial do paciente, devem ser consideradas medidas para prevenção de novos eventos e redução da recorrência a longo prazo.

### 8.1. Terapia Farmacológica

O tratamento médico otimizado da doença cardíaca de base é fundamental para a prevenção de episódios de TE. No caso de pacientes com insuficiência cardíaca com fração de ejeção reduzida, as recomendações da Diretriz Brasileira de Insuficiência Cardíaca Crônica e Aguda incluem tratamento com inibidores da enzima conversora de angiotensina, bloqueadores dos receptores da angiotensina II ou inibidor da neprisilina e do receptor da angiotensina, antagonistas dos receptores mineralocorticoides, betabloqueadores e inibidores do cotransportador 2 da glicose sódica, visando à redução da mortalidade por insuficiência cardíaca e morte súbita.^117^ A realização de cineangiocoronariografia deve ser considerada em pacientes que se apresentam com dor torácica, alterações do segmento ST ou alteração significativa nos níveis de troponina.

Os medicamentos antiarrítmicos, além de seu uso para estabilização aguda da TE, também podem ser considerados para prevenção de recorrências. Em ensaio clínico randomizado, a combinação de amiodarona e betabloqueador foi superior ao uso isolado de betabloqueador ou sotalol na redução de choques pelo CDI.^118^ No entanto, houve significativa taxa de descontinuidade dos medicamentos por efeitos adversos. Em algumas situações mais raras, há indicação de medicamentos específicos para prevenção de TE, como mexiletina na SQTL tipo 3 e quinidina na síndrome de Brugada.^2^

Em pacientes com história de TE, deve haver vigilância rigorosa em relação ao uso de qualquer medicamento com potencial efeito pró-arrítmico ou risco de causar distúrbios metabólicos e eletrolíticos que possam ser gatilhos para TE.

### 8.2. Cardioversor-Desfibrilador Implantável

A TE pode ser a manifestação clínica inicial de arritmia ventricular. Em pacientes com TE com instabilidade hemodinâmica ou reanimados de parada cardiorrespiratória, na ausência de causa reversível, o implante de CDI está indicado como estratégia de prevenção secundária.^59^ Dados de metanálise mostram que o implante do CDI nesses pacientes está associado à redução da mortalidade arrítmica e total.^119^ Na avaliação da indicação de CDI, devem ser levadas em consideração a presença de comorbidades, a qualidade de vida e a expectativa de vida. É importante ressaltar que, na TE, o implante de CDI não deve ser realizado enquanto houver quadro de arritmia incessante, sem controle clínico adequado.

### 8.3. Ablação por Cateter

A ablação por cateter pode ser considerada tanto para estabilização aguda do paciente com TE como para a prevenção de recorrências a longo prazo.

Em pacientes com doença cardíaca isquêmica portadores de CDI, ensaios clínicos randomizados apontam que a ablação por cateter é superior à terapia antiarrítmica isolada para a prevenção de novos episódios de terapia do CDI.^120-123^

Dados de registros mostram que pacientes com TE encaminhados para ablação são mais velhos, têm menor fração de ejeção e maior prevalência de comorbidades em relação aos pacientes sem TE.^73^ Em relação aos resultados da ablação em pacientes com TE, dados de registros mostram sobrevida livre de TV em 60 meses de 54%, não havendo diferença significativa entre pacientes com etiologia isquêmica ou não isquêmica.^73,78^ O sucesso agudo do procedimento com ausência de indução de taquiarritmias ventriculares foi associado a maior sobrevida. A taxa de complicações descritas variou de 3 a 7%.^12,14^ Em registros com menor número de pacientes, a ablação também se mostrou eficaz em pacientes com TE e cardiomiopatia arritmogênica do ventrículo direito ou doença de Chagas.^124^

### 8.4. Modulação Autonômica

A modulação autonômica pode ser considerada para a estabilização aguda e para a prevenção de recorrências a longo prazo em pacientes com TE. A simpatectomia cirúrgica esquerda ou bilateral demonstrou reduzir significativamente a recorrência de episódios de choques pelo CDI em séries de casos.^16^

## 9. Novas Perspectivas

A TE é uma emergência médica ameaçadora à vida e de prognóstico sombrio. Sua abordagem terapêutica é desafiadora e embasada predominantemente em estudos retrospectivos, sendo urgente a necessidade de novas formas de tratamento. Um dos fatores que contribuem para a dificuldade no tratamento da TE é seu grande espectro de condições cardíacas subjacentes. Pode ocorrer em associação a doenças cardíacas estruturais, síndromes arrítmicas genéticas e até mesmo em pacientes com coração normal. Essa diversidade leva a características eletrofisiológicas heterogênicas, com mecanismos arrítmicos distintos. No entanto, um ponto em comum nesses casos é o importante papel do sistema nervoso autônomo na gênese e manutenção da arritmia ventricular. O estudo *Temperature-Related Incidence of Electrical Storm* (TEMPEST)^18^ demonstra que a TE ocorre predominante durante as horas e os dias de trabalho, sugerindo uma variação circadiana na sua distribuição e um papel do tônus simpático na sua ocorrência.

Para combater o estado hiperadrenérgico, a sedação com opioides ou hipnóticos, como o midazolam ou propofol, podem contribuir para a redução dos eventos arrítmicos. Em estudo com 15 pacientes durante uma TE, a sedação profunda com propofol foi capaz de resolver a arritmia ventricular em 80% dos casos.^81^

A neuromodulação é uma forma de tratamento emergente para arritmias ventriculares. A denervação simpática cardíaca reduz a recorrência de TV e choques pelo CDI em pacientes com TE a curto e a longo prazo. Em 1 ano, 58% dos pacientes ficaram livres de choques e não apresentaram recorrência da arritmia ventricular.^125^ Outras formas de modulação autonômica, como anestesia epidural-torácica e denervação renal, estão sendo estudadas, com resultados promissores.^41,126^

Para pacientes instáveis sem resposta ao tratamento convencional, a ECMO pode ser aplicada até um tratamento mais efetivo ser instituído. Entretanto, a taxa de sucesso dessa prática fica em torno de 50%, sugerindo que novos algoritmos de tratamento devem ser testados.^94^

Por fim, a ablação estereotáxica está emergindo como modalidade alternativa para pacientes em TE. A radioterapia corpórea estereotáxica é utilizada rotineiramente em oncologia para o tratamento não invasivo de tumores sólidos com alta precisão e eficácia. Atualmente, está sendo testada em casos de arritmia ventricular, com resultados promissores demonstrando redução na recorrência da TV por volta de 70%; entretanto, seu papel na TE é mais restrito devido à ausência de efeito imediato.^127-130^ O mecanismo de sucesso dessa modalidade é atribuído à formação de fibrose induzida pela radiação^131^, que acaba criando um bloqueio na condução cardíaca. Entretanto, a redução da arritmia ventricular ocorre após alguns dias do procedimento, sugerindo que outros mecanismos possam estar envolvidos.^132^

## Data Availability

os conteúdos subjacentes ao texto do Posicionamento estão contidos no manuscrito.
