# Supplementary material for: Posicionamento sobre Diagnóstico e Tratamento da Tempestade Elétrica – 2026
Source: Arq Bras Cardiol. 2026 Apr 28;123(4):e20260215. [Article in Portuguese] doi: 10.36660/abc.20260215 (PMC13155779; doi:10.36660/abc.20260215)
Supplement: Translation

# Translation: Position Statement on the Diagnosis and Treatment of Electrical Storm – 2026

**DOI:** 10.36660/abc.20260215i

**Authors:** Alexandre de Matos Soeiro, Cristiano Faria Pisani, João Luiz Fernandes Petriz, Bruno Ferraz de Oliveira Gomes, Cláudio Bittencourt das Virgens, José Roberto de Oliveira Silva, Paulo Rogério Soares, Tatiana de Carvalho Andreucci Torres Leal, Alexsandro Alves Fagundes, Luciana Vidal Armaganijan, Ana Luiza Moreno, Francisco Carlos da Costa Darrieux, Luciana Sacilotto, Maurício Pimentel, Fátima Dumas Cintra

**Affiliations:** 1 São Paulo SP Brazil Instituto do Coração do Hospital das Clínicas da Faculdade de Medicina da Universidade de São Paulo, São Paulo, SP – Brazil; 2 São Paulo SP Brazil Hospital das Clínicas da Faculdade de Medicina da Universidade de São Paulo, São Paulo, SP – Brazil; 3 Rio de Janeiro RJ Brazil Hospital Barra D’Or, Rio de Janeiro, RJ – Brazil; 4 Salvador BA Brazil Hospital Universitário Professor Edgard Santos da Universidade Federal da Bahia, Salvador, BA – Brazil; 5 Salvador BA Brazil Universidade do Estado da Bahia, Salvador, BA – Brazil; 6 São Paulo SP Brazil Hospital Sírio Libanês, São Paulo, SP – Brazil; 7 Salvador BA Brazil Universidade do Estado da Bahia, Salvador, BA – Brazil; 8 São Paulo SP Brazil Instituto Dante Pazzanese de Cardiologia, São Paulo, SP – Brazil; 9 Rio de Janeiro RJ Brazil Instituto D’Or de Pesquisa e Ensino, Rio de Janeiro, RJ – Brazil; 10 Porto Alegre RS Brazil Hospital de Clínicas de Porto Alegre, Porto Alegre, RS – Brazil; 11 São Paulo SP Brazil Universidade Federal de São Paulo, São Paulo, SP – Brazil

**Table:** 

Position Statement on the Diagnosis and Treatment of Electrical Storm – 2026	
The report below lists declarations of interest as reported to the SBC by the experts during the period of the development of these statement, 2025/2026.	
Expert	Type of relationship with industry
Alexandre de Matos Soeiro	Financial declaration B - Research funding under your direct/personal responsibility (directed to the department or institution) from the brazilian or international pharmaceutical, orthosis, prosthesis, equipment and implants industry:- Biomerieux.
Alexsandro Alves Fagundes	Nothing to be declared
Ana Luiza Moreno de Almeida Silva	Nothing to be declared
Bruno Ferraz de Oliveira Gomes	Nothing to be declared
Claudio Marcelo Bittencourt das Virgens	Nothing to be declared
Cristiano Faria Pisani	Financial declaration A - Economically relevant payments of any kind made to (i) you, (ii) your spouse/partner or any other person living with you, (iii) any legal person in which any of these is either a direct or indirect controlling owner, business partner, shareholder or participant; any payments received for lectures, lessons, training instruction, compensation, fees paid for participation in advisory boards, investigative boards or other committees, etc. from the brazilian or international pharmaceutical, orthosis, prosthesis, equipment and implants industry: - Johnson & Johnson.
Fatima Dumas Cintra	Other relationships Any economically relevant equity interest in companies in the healthcare or education industry or in any companies competing with or supplying to SBC:- Health area.
Francisco Carlos da Costa Darrieux	Nothing to be declared
João Luiz Fernandes Petriz	Financial declaration A - Economically relevant payments of any kind made to (i) you, (ii) your spouse/partner or any other person living with you, (iii) any legal person in which any of these is either a direct or indirect controlling owner, business partner, shareholder or participant; any payments received for lectures, lessons, training instruction, compensation, fees paid for participation in advisory boards, investigative boards or other committees, etc. from the brazilian or international pharmaceutical, orthosis, prosthesis, equipment and implants industry: - Servier: Vastarel.
José Roberto de Oliveira Silva Filho	Financial declaration A - Economically relevant payments of any kind made to (i) you, (ii) your spouse/partner or any other person living with you, (iii) any legal person in which any of these is either a direct or indirect controlling owner, business partner, shareholder or participant; any payments received for lectures, lessons, training instruction, compensation, fees paid for participation in advisory boards, investigative boards or other committees, etc. from the brazilian or international pharmaceutical, orthosis, prosthesis, equipment and implants industry: - Chiesi: Trimbow; GSK: Vaccines; Libbs: ticagrelor, ramipril and candesartan; Biolab: vasopressin. Other relationships Funding of continuing medical education activities, including travel, accommodation and registration in conferences and courses, from the brazilian or international pharmaceutical, orthosis, prosthesis, equipment and implants industry: - Daiichi Sankyo: prasugrel.
Luciana Armaganijan	Nothing to be declared
Luciana Sacilotto	Nothing to be declared
Maurício Pimentel	Financial declaration A - Economically relevant payments of any kind made to (i) you, (ii) your spouse/partner or any other person living with you, (iii) any legal person in which any of these is either a direct or indirect controlling owner, business partner, shareholder or participant; any payments received for lectures, lessons, training instruction, compensation, fees paid for participation in advisory boards, investigative boards or other committees, etc. from the brazilian or international pharmaceutical, orthosis, prosthesis, equipment and implants industry: - Libbs: atrial fibrillation.
Paulo Rogério Soares	Nothing to be declared
Tatiana de Carvalho Andreuci Torres Leal	Other relationships Funding of continuing medical education activities, including travel, accommodation and registration in conferences and courses, from the brazilian or international pharmaceutical, orthosis, prosthesis, equipment and implants industry: - Daiichi Sankyo: prasugrel.

## Table of contents

**1. Introduction** 4
  **1.1. Objectives of this Position Statement** 4
  **1.2. Methods** 4
**2. Pathophysiology** 5
**3. Initial approach in the Emergency Department** 6
  **3.1. Diagnosis** 6
  **3.2. Electrocardiography** 7
  **3.3. Laboratory Tests and Biochemical Evaluation** 7
  **3.4. Evaluation of the Implantable Cardioverter-Defibrillator** 7
  **3.5. Initial Stabilization Measures** 7
**4. Pharmacological Therapy** 8
  **4.1. Antiarrhythmic Agents** 8
  **4.2. Amiodarone** 9
  **4.3. Lidocaine** 9
  **4.4. Adrenergic Beta-Blockers** 10
  **4.5. Magnesium Sulfide** 10
**5. Management and Programming of the Implantable Cardioverter-Defibrillator** 11
  **5.1. Implantable Cardioverter-Defibrillator Therapies** 11
  **5.2. Event Detection** 11
  **5.3. Shock Therapy** 13
  **5.4. Antitachycardia Pacing** 13
  **5.5. Artificial Cardiac Pacing** 13
  **5.6. Management of Patients with an Implantable Cardioverter-Defibrillator During Electrical Storm** 13
**6. Catheter Ablation in the Setting of Electrical Storm** 14
  **6.1. Additional Therapies for Electrical Storm** 16
    **6.1.1. Sedation** 16
    **6.1.2. Thoracic Epidural Anesthesia** 17
    **6.1.3. Stellate Ganglion Block** 17
    **6.1.4. Surgical Cardiac Sympathetic Denervation** 17
    **6.1.5. Renal Sympathetic Denervation** 17
    **6.1.6. Circulatory Management** 17
    **6.1.7. Extracorporeal Membrane Oxygenation** 17
    **6.1.8. Intra-Aortic Balloon Pump** 18
    **6.1.9. TandemHeart Circulatory Support and Impella Device** 18
    **6.1.10. Device Selection** 18
**7. Management of Electrical Storm Associated with Channelopathies** 18
**8. Long-Term Prevention of Recurrence** 20
  **8.1. Pharmacotherapy** 20
  **8.2. Implantable Cardioverter-Defibrillator** 20
  **8.3. Catheter Ablation** 21
  **8.4. Autonomic Modulation** 21
**9. New Perspectives** 21
**References** 22

## 1. Introduction

Electrical storm (ES) is defined as a state of cardiac electrical instability characterized by three or more episodes of sustained ventricular tachycardia (VT), ventricular fibrillation (VF), or appropriate shocks from an implantable cardioverter-defibrillator (ICD) within 24 hours, with episodes separated by at least 5 minutes (Central Illustration).^1,2^

ES occurs in 4% to 40% of patients with an ICD, with a median of two episodes per patient and a higher incidence during the first days following device implantation. Patients who, in addition to ventricular arrhythmias, experience repetitive episodes (whether or not formally classified as ES) have higher mortality, which is directly proportional to the number of episodes occurring over a shorter time interval.^3^

The incidence of ES is lower in patients with an ICD for primary prevention. In a subanalysis of 719 patients from the Multileft Automatic Defibrillator Implantation Trial II (MADIT-II), which evaluated prophylactic implantation of an ICD in patients with prior acute myocardial infarction (AMI) and left ventricular ejection fraction (LVEF) ≤ 30%, the incidence of ES was 4.4% over a mean follow-up of 20.6 months.^4-8^ In addition to the presence of an ICD, other risk factors are commonly identified in patients presenting with ES to the emergency department, including reduced ejection fraction, chronic kidney disease, and prior AMI.^6^

In patients with chronic Chagas heart disease, a recently published meta-analysis reported an annual ES incidence of 9.1%.^9-11^ In another study, the occurrence of repetitive shocks, particularly more than four per month, was associated with increased mortality in patients with chronic Chagas heart disease, with a mean life expectancy of 2.1 months.^12^

ES is a clinical condition associated with high mortality, reaching up to 14% within the first 48 hours. Even when compared with patients experiencing isolated VT/VF episodes, ES increases the risk of in-hospital death by 2.4-fold and by up to 5.4-fold over the subsequent 3 months. Therefore, it is essential that general practitioners, cardiologists, emergency physicians, and intensivists promptly recognize and appropriately manage ES.^13^

### 1.1. Objectives of this Position Statement

To provide practical guidance for physicians (cardiologists and non-cardiologists) and the multidisciplinary health care team regarding the initial approach, diagnosis, and clinical stabilization of patients with ES, in accordance with best evidence-based practices;

To establish a standardized approach to pharmacological management of patients with ES, based on available evidence and treatment options within the national health care setting;

To describe indications and clinical scenarios for the use of catheter ablation as adjunctive therapy;

To discuss, in light of the available evidence, relevant adjunctive therapies, their indications, and long-term perspectives.

### 1.2. Methods

Recommendations:

- **Class I:** Conditions for which there is conclusive evidence and, failing that, general agreement that a given procedure is safe and useful/effective;
- **Class II:** Conditions for which there is conflicting evidence and/or divergence of opinion about the safety and usefulness/efficacy of the procedure;
- **Class IIa:** Evidence or opinion in favor of the procedure. The majority agrees;
- **Class IIb:** Safety and usefulness/efficacy are less well established, and there is no predominance of opinions in favor of the procedure;
- **Class III:** Conditions for which there is evidence and/or a consensus that the procedure is not useful/effective, and in some cases may be harmful.

Evidence:

- **Level A:** Data obtained from several large randomized studies, studies with similar results, and/or robust meta-analysis of randomized controlled trials;
- **Level B:** Data obtained from a less robust meta-analysis, a single randomized study, or from nonrandomized (observational) studies;
- **Level C:** data obtained from consensual expert opinions.

## 2. Pathophysiology

ES may occur in a wide range of clinical conditions, including AMI, ischemic or nonischemic cardiomyopathy, and in the presence of inherited arrhythmia syndromes (such as Brugada syndrome [BrS] and short QT syndrome [SQTS]).^14^ When a patient presents with ES, potential triggers and underlying substrates should be systematically investigated (Figure 1). These substrates and mechanisms are directly involved in the pathophysiology of ES.^15^

**Figure 1:** Contribution of predisposing substrates and mechanisms to the pathophysiology of electrical storm generation. VF: ventricular fibrillation; VT: ventricular tachycardia. Adapted from Looi et al.^15^

For ES occurrence, an interplay between the autonomic nervous system and a predisposing electrophysiological substrate is crucial. This interplay evolves over time as a consequence of myocardial scarring, ischemia, or ventricular dilatation secondary to progression of heart failure (HF). Therefore, the identification of triggers and predictors is essential to recognize patients at higher risk, with the aim of reducing both the risk and the occurrence of ES.

However, in most cases, no clear cause for the development of ES can be identified. In the Shock Inhibition Evaluation With Azimilide (SHIELD) study, an ES trigger was identified in only 13% of patients.^7^ Similarly, risk factors for ES may be difficult to determine. In a study involving patients with an ICD, the variables most strongly associated with ES were reduced ejection fraction and the use of class IA antiarrhythmics.^16^

Structural heart diseases that may predispose to ES include myocardial fibrosis, fatty infiltration, or even small areas of myocardial scarring. In such cases, the substrate may give rise to reentrant or focal VT. Abnormal automaticity or triggered activity may be correlated with the initiation and maintenance of the tachyarrhythmia. Nevertheless, the specific factors that determine the occurrence of ES are not yet fully understood.

The majority of ES cases consist of monomorphic VT (MVT). In a meta-analysis of patients with ES, 94% had underlying structural heart disease (ischemic or nonischemic), and 80% presented with MVT related to a myocardial scar-based mechanism.^17^ In contrast, polymorphic VT (PVT) and VF are more frequently observed in the setting of AMI and advanced HF.

The autonomic nervous system plays a pivotal role in the modulation of arrhythmogenesis. In the context of ES, sympathetic tone is increased in patients with structural heart disease. In the Temperature-Related Incidence of Electrical Storm (TEMPEST) study, in which 83% of patients had some form of cardiomyopathy, the prevalence of ES was higher on weekdays and during working hours.^18^ In certain inherited cardiomyopathies and channelopathies, such as long QT syndrome (LQTS) and catecholaminergic VT, arrhythmic events are typically triggered by physical or emotional stress.

Several molecular mechanisms have been proposed to explain the arrhythmogenesis of ES. The primary mechanism involves alterations in the phosphorylation of proteins that regulate calcium handling, leading to intracellular calcium imbalance within cardiomyocytes. This imbalance promotes arrhythmogenic triggers mediated by afterdepolarizations, hypocontractility resulting from calcium depletion in the sarcoplasmic reticulum, and triggered activity.

Although multiple mechanisms have been proposed, significant uncertainty remains regarding the comprehensive pathophysiological understanding of ES. Further studies are required to better elucidate this complex clinical entity.

## 3. Initial Approach in the Emergency Department

### 3.1. Diagnosis

The initial evaluation in the Emergency Department begins with the recognition of patients at risk for developing ES. Most patients presenting with ES in the emergency setting have underlying structural heart disease, such as HF with reduced LVEF (of ischemic or nonischemic etiology), arrhythmogenic right ventricular (RV) cardiomyopathy, sarcoidosis, amyloidosis, Chagas disease, or channelopathies, including BrS.^19,20^

The first step in the assessment of a patient with suspected ES is to determine the presence of hemodynamic instability; if present, management should follow current Advanced Cardiovascular Life Support (ACLS) guidelines.^21,22^ Patients with ventricular arrhythmias and pulselessness should undergo immediate defibrillation. Electrolyte abnormalities must be promptly corrected, with particular attention to hypomagnesemia and hypokalemia. The hemodynamics team should be activated if myocardial ischemia is suspected, especially in the presence of ST-segment elevation. When ES is precipitated by acute decompensated HF, hemodynamic support with vasoactive agents and, when indicated, mechanical circulatory support devices should be instituted. These patients should be admitted to an intensive care unit for ongoing monitoring and management of complications.

After initial stabilization, potentially reversible causes of ES must be actively investigated, including drug toxicity, decompensated HF, new or ongoing myocardial ischemia, thyrotoxicosis, infections, fever, and electrolyte disturbances, particularly hypokalemia and hypomagnesemia.^4^ A thorough review of medications that may prolong the QT interval is essential, including antiarrhythmic agents, which, although beneficial in several clinical scenarios, may also increase QT duration, such as amiodarone.^16^

The clinical presentation of an ES episode is highly variable and may include palpitations, chest pain, syncope, acute decompensated HF, hypotension, or cardiac arrest.^18,23^ Presentation depends on multiple factors, including heart rate, the degree of ventricular dysfunction, underlying cardiac disease, and the presence of an ICD. Patients with more severe ventricular dysfunction may poorly tolerate ES. In some cases, patients may present in a relatively stable condition with a lower ventricular rate, characterized as slow VT, typically in the range of 100 to 120 beats per minute.

### 3.2. Electrocardiography

All patients with suspected es should undergo a 12-lead electrocardiography (ECG) and be placed on continuous cardiac monitoring.

Most episodes of ES present as MVT, typically secondary to myocardial scar-related reentrant mechanisms. In contrast, PVT and VF are more commonly observed in the setting of acute ischemia, electrolyte disturbances, QT interval prolongation, and inherited channelopathies.

In some cases, distinguishing supraventricular tachycardia with aberrant conduction from VT can be challenging. For this reason, several diagnostic algorithms have been developed over recent years to aid in the differentiation of wide QRS complex tachycardias.^24-26^ In extreme situations characterized by hemodynamic instability and imminent risk of death, wide-complex tachycardias should be promptly treated as ventricular arrhythmias.

### 3.3. Laboratory Tests and Biochemical Evaluation

A comprehensive laboratory evaluation should include a complete blood count, high-sensitivity cardiac troponin, B-type natriuretic peptide, thyroid function tests (thyroid-stimulating hormone and free thyroxine), serum electrolytes (sodium, potassium, calcium, and magnesium) as well as additional tests guided by the suspected etiology, such as renal function markers and C-reactive protein.

### 3.4. Evaluation of the Implantable Cardioverter-Defibrillator

In patients with an ICD who have received device therapies, the first step after clinical stabilization is to determine whether the delivered therapy was appropriate. Inappropriate ICD therapies may occur in up to 40% of cases, despite advances in ventricular arrhythmia detection algorithms.^27^

Device interrogation allows for detailed assessment of the number and timing of ventricular arrhythmia episodes, heart rate, arrhythmia morphology, mode of onset, the ICD's response to these arrhythmias (such as antitachycardia pacing [ATP] or shock), and the effectiveness of delivered therapies. It is important to emphasize that patients experiencing frequent ICD shocks are at increased risk of developing associated psychiatric disorders, including anxiety and depression.

The most common causes of inappropriate ICD therapies include supraventricular arrhythmias falling within the programmed VT/VF detection zones (e.g., atrial fibrillation with rapid ventricular response), ventricular lead oversensing (such as T-wave oversensing), and ventricular lead malfunction due to conductor fracture or insulation failure.^28^

A chest radiograph should be obtained in all patients with suspected inappropriate ICD therapy to evaluate for lead displacement or lead fracture.

### 3.5. Initial Stabilization Measures

The traditional ACLS-guided approach to wide QRS complex tachyarrhythmias is often suboptimal for patients experiencing ES, given the unique pathophysiological features of this condition.^29^ During ES, sympathetic tone is markedly increased and may be further exacerbated by repetitive ICD shocks, sometimes necessitating temporary deactivation of device therapies and the adoption of alternative approaches. This catecholamine surge and heightened sympathetic activation increase myocardial susceptibility to ischemia and recurrent arrhythmias, thereby perpetuating the ES state.^30^

In this context, suppression of the sympathetic system through the use of beta-blockers plays a central role in the management of ES. A study involving patients with prior AMI and ES demonstrated that beta-blocker therapy was associated with a reduction in the incidence of sudden cardiac death.^31,32^

#### 3.5.1. Beta-Blockers

Several studies have sought to clarify the role of beta-blockers in ES and their effectiveness in reducing sympathetic tone. Experimental studies have demonstrated that nonselective beta-blockers reduce myocardial oxygen consumption while increasing oxygen supply, thereby attenuating myocardial injury and modifying electrical thresholds for ventricular arrhythmias.^33^ Multiple beta-blockers have been investigated in this setting, including propranolol, a nonselective, lipophilic agent capable of crossing the blood-brain barrier, and cardioselective beta-blockers such as esmolol, which can be administered and titrated via continuous intravenous (IV) infusion.^34^ In a randomized clinical trial comparing propranolol at a dose of 40 mg every 6 hours with metoprolol, propranolol was associated with greater efficacy in stabilizing ES.^35^

#### 3.5.2. Amiodarone and Other Antiarrhythmic Agents

Although several antiarrhythmic agents have been described for the treatment of ES, amiodarone remains the first-line pharmacological therapy.^13,36^ Other agents, such as class I antiarrhythmics (e.g., lidocaine), are not recommended as first-line therapy for ES due to their variable efficacy in this setting. They may be effective in selected cases of ES secondary to myocardial ischemia, but have been shown to be inferior to amiodarone in patients with refractory VF.^37,38^ Outside the context of ischemia, lidocaine demonstrates a low arrhythmia conversion rate, with reported success rates ranging from approximately 8% to 30%.^39^

Pharmacological therapy for the treatment of ES will be addressed in a separate section of this document.

#### 3.5.3. Sympathetic Block

During episodes of ES, patients are exposed to substantial physical and emotional stress, further increasing sympathetic nervous system activity and perpetuating ventricular arrhythmias. Measures aimed at stress reduction should therefore be considered, such as placing the patient in a calm environment and administering low doses of anxiolytic agents, including benzodiazepines.

Studies suggest that sympathetic nervous system activity can be reduced through the administration of sedation boluses, particularly with propofol, due to its sympatholytic properties.^40^ In refractory cases, endotracheal intubation and deep sedation should be considered, even when using alternative sedative agents such as midazolam.

Two additional, less invasive options include thoracic epidural anesthesia, an alternative therapy that directly inhibits cardiac sympathetic innervation without the potential adverse effects of deep sedation,^41^ and stellate ganglion block, which can be performed via a paratracheal approach under bedside ultrasound guidance.^29,42^

#### 3.5.4. Management and Programming of the Implantable Cardioverter-Defibrillator

In patients with an ICD, verifying the occurrence of device therapies and determining whether they were appropriate is a fundamental step in the initial evaluation. When inappropriate therapies are identified, deactivation of ICD shock therapy should precede any other intervention.^43^ In situations in which access to device programmers is limited, placement of a magnet over the ICD may be used to temporarily suspend both ATP and shock therapies. Inappropriate therapies induce a hyperadrenergic state, which in itself may precipitate ES. For the same reason, even when ICD therapies are appropriate, temporary deactivation of shock therapies may be considered in hemodynamically stable patients.^28^

In cases of appropriate shocks for MVT during ES, adjustments to detection parameters and optimization of therapy programming may be attempted. ATP is a safe and effective strategy and is associated with less discomfort for the patient.^44,45^

In some circumstances, device pacing algorithms themselves may be proarrhythmic. ICD programming and management strategies will be discussed in a dedicated section of this document.

#### 3.5.5. Specific Scenarios

Finally, there are two clinical situations in which the management of ES represents an exception to the general principles outlined above. The first involves patients with a history of BrS or a high level of suspicion based on prior ECG findings. The incidence of ES in patients with BrS is unknown; however, these patients are at increased risk of developing potentially fatal arrhythmias.^46^

Many pharmacological interventions traditionally used in ES are ineffective in this population or may further exacerbate ES in patients with BrS. In this setting, isoproterenol, a beta-adrenergic agonist, should be considered first-line therapy. This agent is also effective in the treatment of ES in patients with SQTS.^47^

The second exception occurs when the presenting rhythm is PVT, which accounts for 7% of ES cases and may represent the first manifestation of myocardial ischemia.

Recurrent episodes of PVT should raise suspicion for acquired LQTS, and management requires a specific approach to prevent degeneration into *torsades de pointes* (TdP). Acquired QT interval prolongation is most commonly related to polypharmacy, with or without associated electrolyte disturbances, such as hypokalemia and hypomagnesemia. In this context, amiodarone should be avoided, and all other medications known to prolong the QT interval, including antibiotics, should be discontinued. Cor rection of electrolyte disturbances is mandatory. In cases of QT-related arrhythmias associated with bradycardia, bradycardia should be avoided; temporary transvenous pacing may be considered to prevent bradycardia and allow for overdrive pacing.^48^

Figure 2 summarizes the main strategies involved in the initial management of ES.

**Figure 2:** Algorithm proposed for the treatment of electrical storm. CCB: calcium channel blockers; EPS: electrophysiological study; IABP: intra-aortic balloon pump; MVT: monomorphic VT; PVCs: premature ventricular contractions; PVT: polymorphic ventricular tachycardia; VF: ventricular fibrillation. Adapted from Eifling et al.^23^

## 4. Pharmacological Therapy

Pharmacological therapy is primarily based on four pillars: the use of antiarrhythmic agents, beta-blockers, sedation management, and hemodynamic support.

It is important to emphasize that clinical management also includes the identification of potential triggering factors and their prompt correction. Myocardial ischemia, decompensated HF, low cardiac output with pulmonary congestion, thyrotoxicosis, electrolyte disturbances, drug toxicity and proarrhythmic medications, as well as increased adrenergic tone, may favor both the occurrence and recurrence of ventricular arrhythmias and ES.

### 4.1. Antiarrhythmic Agents

Antiarrhythmic agents act on myocardial ion channels, allowing arrhythmia termination in certain situations or facilitating electrical cardioversion, primarily by inhibiting triggers that reinitiate arrhythmias. In addition, after arrhythmia termination, antiarrhythmic agents play a central role in preventing the recurrence of ventricular arrhythmias.^1,2,49^

According to their mechanisms of action, antiarrhythmic agents are categorized by the Vaughan–Williams classification^50^ (Table 1). Antiarrhythmics available in Brazil are also listed in Table 1.^4^

**Table 1:** Vaughan–Williams classification of antiarrhythmics

Class	Pharmacodynamic action	Medications available in Brazil
IA	Moderate blockade of Na^+^ and K^+^ currents Intermediate binding and dissociation kinetics May prolong QRS and QTc	—
IB	Decrease of electrical excitability Mild blockade of fast and late Na^+^ currents Rapid binding and dissociation kinetics Does not prolong QTc	Lidocaine Phenytoin
IC	Intense blockade of fast and late Na^+^ currents with mild blockade of Ca^2+^ currents and beta-adrenergic receptors Slow binding and dissociation kinetics Prolongs QRS	Propafenone
II	Beta-blockers	Propranolol Metoprolol succinate Metoprolol tartrate Atenolol
III	Blockade of repolarizing K^+^ channels Prolongation of the refractory period, impairing reentry Prolongs the QT interval	Amiodarone Sotalol
IV	Ca^2+^ channel blockade	Verapamil Diltiazem

The antiarrhythmic agents most commonly used in the emergency management of ES are amiodarone and lidocaine, primarily due to the availability of IV infusion formulations.

### 4.2. Amiodarone

IV amiodarone is the first-line medication for the treatment of ES.^51^ It is also the most effective antiarrhythmic agent for controlling ventricular arrhythmias in patients with ventricular dysfunction and/or structural heart disease, with a classic study reporting a conversion rate of approximately 60%.^52^

Amiodarone has a slow onset of action; therefore, a loading dose administered at a higher infusion rate is required, followed by continuous infusion to achieve adequate tissue saturation and the expected therapeutic effect. In clinical practice, an initial IV loading dose of 6 to 7 mg/kg (approximately 300 mg) is administered, with an infusion duration ranging from 20 to 60 min, depending on the absence or presence of ventricular dysfunction, respectively. The maintenance dose ranges from 900 to 1,200 mg/day, administered for 24 or 48 h via continuous infusion pump. In selected cases, the maximum daily dose may reach 2.2 g. IV administration may frequently induce hypotension, depending on the dose and infusion rate, and caution is required in patients with low cardiac output or hemodynamic instability.^21,22^ After this period, therapy should be transitioned to the oral route, usually maintained at 600 mg/day until a cumulative dose of 10 g is achieved, which is considered adequate loading.

Amiodarone has clinically relevant adverse effects that should not be overlooked. It can prolong the QT interval and may therefore induce proarrhythmic effects. Consequently, it is contraindicated in patients with ES associated with LQTS or PVT. During the loading phase, daily 12-lead ECG monitoring should be considered to assess the QT interval. If QT prolongation beyond 500 ms is observed, dose reduction or medication discontinuation should be considered. Chronic use is associated with corneal deposits and photosensitivity (25%-75% of patients), elevation of liver enzymes (30%), hepatitis, cirrhosis (< 3%), pulmonary fibrosis, and thyroid dysfunction.^53^

### 4.3. Lidocaine

When amiodarone is ineffective or contraindicated, lidocaine is considered a second-line agent, either in combination with amiodarone or as monotherapy. Although its efficacy is lower than that of amiodarone, lidocaine is safe and relatively better tolerated in patients with myocardial ischemia. It can also be combined with other medications that prolong the QT interval. The most common adverse effects are neurological and include agitation, anxiety, confusion, lethargy, loss of consciousness, paresthesia, psychosis, seizures, and slurred speech.^54^ Less common adverse effects include reduced airway sensitivity due to depression of protective reflexes and decreased calcium influx in smooth muscle.^55^

A loading dose of 1-1.5 mg/kg is administered over approximately 30 min, followed by a maintenance infusion of 1 to 4 mg/min.^4^

### 4.4. Adrenergic Beta-Blockers

Adrenergic stimulation plays an undisputed role in the pathophysiology of ES. Accordingly, the addition of an adrenergic beta-blocker enhances the efficacy of antiarrhythmic therapy by reducing the recurrence of ventricular arrhythmias. However, their use is limited in patients presenting with low cardiac output, refractory congestion, or ongoing vasoactive drug therapy, as beta-blockers may precipitate or worsen cardiogenic shock.^48^

IV esmolol may be considered because of its rapid onset of action, short half-life, and ease of titration, and has demonstrated favorable results.^34^ Nevertheless, oral beta-blockers remain the preferred option whenever feasible. Initiation or optimization of the patient's baseline beta-blocker dose should be considered in all ES scenarios. Particular attention should be paid to dosing and tolerance of carvedilol, given its additional hypotensive effect related to concomitant alpha-1 adrenergic blockade.^29^

In addition to beta-blockers commonly used in patients with ventricular arrhythmias and ventricular dysfunction, such as carvedilol and metoprolol, propranolol has shown promising results. Propranolol is a nonselective beta-1 and beta-2 adrenergic blocker and also exerts central nervous system effects due to its lipophilic properties. Thus, beyond its antiarrhythmic effect, it provides anxiolytic benefits and acts on sodium, potassium, and calcium channels in cardiac cells, reducing QT interval dispersion.^14,15^

A randomized clinical trial compared propranolol 40 mg every 6 h with metoprolol 50 mg every 6 h, both administered concomitantly with amiodarone infusion, in patients with an ICD and ES. Patients treated with propranolol showed a reduction in the number of VT episodes (incidence rate ratio, 0.375) and ICD therapies (incidence rate ratio, 0.428). At the end of the first 24 h of treatment, 90% of patients in the propranolol group were free of ES, compared with 53.3% in the metoprolol group. Importantly, despite the negative inotropic effects of propranolol, the medication was well tolerated.^35^

### 4.5. Magnesium Sulfide

Magnesium is the second most prevalent intracellular ion and plays a critical role in the myocardium. It regulates ion flux through sodium, potassium, and calcium channels during electrical activity and excitation–contraction coupling. IV magnesium sulfate has antiarrhythmic properties and is typically administered at a dose of 2-3 g.

The use of magnesium in patients with ES is unequivocal in the setting of PVT and QT interval prolongation, even when serum magnesium levels are within the normal range.^1,2,21,22,43^ Despite its well-established role in this context, there is limited evidence supporting its efficacy in patients with MVT. Therefore, magnesium sulfate is not indicated for arrhythmia termination but rather as an adjunctive therapy.

Approximately 38% of patients with ES present with serum magnesium deficiency, and 72% have risk factors that increase magnesium loss, such as the use of high-dose diuretics. Correction and maintenance of serum magnesium levels above 2 mg/dL reduce the burden of premature ventricular complexes as well as episodes of nonsustained VT, thereby decreasing ES recurrence.^56^

Table 2 presents the recommended pharmacological treatment strategies for the management of ES.

**Table 2:** Recommended pharmacological treatment strategies for the management of electrical storm

Line of therapy	Antiarrhythmics	Adrenergic blockers	Adjunctive therapies
First line	Amiodarone: Bolus 300 mg (approximately 6-7 mg/kg) over 20-60 min Maintenance 900-1,200 mg/day for 24 h Repeat 150 mg if recurrent ventricular arrhythmia	Oral beta-blockers: Propranolol 40 mg every 6 h (preferred) Metoprolol 25-50 mg every 12 h (with planned dose escalation) Optimization of chronic carvedilol and bisoprolol doses	IV magnesium sulfate: 2-3 g over 30 min (mandatory in patients with PVT) Magnesium replacement to maintain adequate serum levels (> 2 mEq/L)
Second line	IV lidocaine: Bolus 1-1.5 mg/kg; maintenance infusion 1-7 µg/min	Beta-blockers: Esmolol bolus 0.5 mg/kg followed by continuous infusion of 50-300 µg/kg/min	Potassium chloride IV or oral: Replacement to maintain adequate serum potassium levels (> 4 mEq/L)

IV: intravenous; VT: polymorphic ventricular tachycardia.

## 5. Management and Programming of the Implantable Cardioverter-Defibrillator

Patients with an ICD may develop ES in 4%-7% of primary prevention cases and in 10% to 28% of secondary prevention cases.^4,57^ Electrical therapy with cardioversion, muscle fatigue caused by recurrent arrhythmias, and the occurrence of apoptosis and autonomic and electrolyte imbalance contribute to a 2- to 3-fold increase in mortality among ICD recipients with ES.^58^ In addition, patients discharged after stabilization of an ES episode show a similarly increased incidence of HF decompensation in the subsequent weeks.^14^ Appropriate adjustment of ICD programming to optimize delivered therapies, maintaining efficacy while avoiding hemodynamic compromise, is therefore fundamental to patient management.^59^

### 5.1. Implantable Cardioverter-Defibrillator Therapies

The automatic therapies available in contemporary ICDs include includes high- or low-energy shocks, pacing at a rate higher than the tachycardia (ATP), and conventional pacing. The latter, similar to that provided by pacemakers, is essential for therapeutic support in patients who develop bradycardia while receiving antiarrhythmics and beta-blockers. Shock therapy in patients with ES may occur in two distinct situations: i) appropriate therapy in patients with sustained, potentially fatal ventricular arrhythmias; or ii) early therapy in patients with arrhythmias that may resolve spontaneously, such as nonsustained VT.^60^ Repetitive shocks delivered to an awake patient are not only highly uncomfortable but may also contribute to worsening ventricular function and further activation of the adrenergic system, thereby perpetuating ES (Figure 3).^61^

**Figure 3:** Recording of an ES episode. The patient received 35 shocks over an 8-hour period. Several shocks occurred sequentially without successful arrhythmia termination. Some VT episodes occurred at a rate of approximately 110 beats per minute (monitoring zone, monomorphic VT) or terminated spontaneously (nonsustained VT). ES: electrical storm; VT: ventricular tachycardia.

### 5.2. Event Detection

Therapies automatically delivered by the ICD can be escalated according to heart rate, which is classified into different detection zones. Depending on the selected programming, more or less aggressive intervention strategies may be chosen. Within the respective detection zones, VT may be defined in one or two different rate zones, as well as VF and a monitoring zone, in which the device can store electrograms of high-rate episodes without delivering shock therapy or ATP. The algorithms used by ICDs detect arrhythmias based on heart rate and the assessment of discriminatory criteria designed to exclude supraventricular arrhythmias. The lower the initial detection rate programmed for therapy delivery, the more aggressive the intervention, with the possibility of shocks even during conditions such as sinus tachycardia. Conversely, programming that includes only very high heart rates as criteria for therapy within the VF zone (shock box) may reduce inappropriate therapies but may preclude automatic intervention during VT episodes with lower rates.

Another important aspect of detection is the number of beats required to complete event counting. The ICD defines a tachyarrhythmia as detected when a predefined number of beats at a given rate is recognized. For example, if the algorithm defines a VT zone as 16 beats at a rate of 160 bpm, the arrhythmia will be detected only after the 16th consecutive beat at or above 160 bpm. The manner in which counting is performed directly affects the interval between arrhythmia onset and delivery of shock or ATP. Other methods for specifying this delay may be used, depending on the detection zone, such as a predefined time in seconds or a minimum number of beats counted within a sample window (X in Y). These strategies are useful for discriminating nonsustained arrhythmias or frequent premature beats. Inappropriate shocks can be reduced by increasing detection time; however, excessive prolongation may compromise patient safety in the presence of unstable arrhythmias.^60^

### 5.3. Shock Therapy

Shock therapy is the most characteristic ICD intervention. Delivered using a biphasic waveform configuration, defibrillation energy is transmitted to the heart through shock coils positioned in the endocardium or, in the case of subcutaneous ICDs, within the subcutaneous tissue.^62^ The effectiveness of shock therapy in terminating a given VT/VF episode depends on the defibrillation threshold.^63,64^ Lower-energy shocks may be programmed for termination of MVT in zones below the VF zone. However, in arrhythmias occurring within the VF zone, although an initial shock of 15 to 25 J may be attempted, escalation of therapy typically involves a sequence of successive shocks with progressively increasing energy up to the maximum output, generally allowing up to six attempts (Figures 4 e 5).^65^

**Figure 4:** Interval plot during an episode of ventricular tachycardia. Atrial rate is dissociated from ventricular rate. An attempt at arrhythmia termination with antitachycardia pacing (ramp) is observed, followed by an ineffective shock (35.9 J) before the effective shock that results in termination of the tachycardia.

**Figure 5:** Intracavitary electrogram recording of ventricular tachycardia (center portion of the image), showing the atrial electrogram (Atip to Aring) and the ventricular electrogram (RVtip to RVring). The appropriate cardioversion/defibrillation shock is effective in restoring rhythm.

### 5.4. Antitachycardia Pacing

Transvenous ICD systems, similar to pacemakers, are equipped with electrodes positioned in the ventricular chamber that allow for artificial ventricular pacing. In this setting, reentrant ventricular arrhythmias can be terminated by delivering pacing impulses at a rate slightly faster than the VT rate (Figure 6). The principle of this therapy is interruption of the reentrant tachycardia circuit. The main advantage of ATP programming is termination of VT episodes without the discomfort and deleterious hemodynamic effects associated with shock therapy.^65^

**Figure 6:** Example of antitachycardia pacing during ventricular tachycardia. Delivery of eight pacing pulses at a rate faster than the tachycardia (overdrive) is observed. Restoration of sinus rhythm occurs without the need for shock therapy.

The Pacing Fast Ventricular Tachycardia Reduces Shock Therapies (PainFREE Rx II) trial evaluated the usefulness of ATP programming for tachycardias with rates up to 250 bpm. VT termination without shock was achieved in 72% of patients with this programming strategy, resulting in a reduction in shocks and improvement in quality of life.^66^

### 5.5. Artificial Cardiac Pacing

Every ICD incorporates pacemaker functionality with the capability for electrical cardioversion. Conventional pacing for bradyarrhythmia control may be required in patients with ventricular arrhythmias and ES. ICD configurations may be single- or dual-chamber and can also be combined with cardiac resynchronization therapy. Accordingly, any patient with an ICD may receive different atrial- or ventricular-based pacing modes (e.g., AAI and DDD). Patients who develop ES often receive aggressive therapy, including pharmacological and nonpharmacological measures that may exacerbate bradycardia. Careful attention to antibradycardia programming is therefore essential to ensure a physiologically appropriate heart rate for the patient's clinical condition. Chronotropic incompetence or concomitant atrioventricular conduction disturbances may further compromise hemodynamic stabilization in patients with ES.^67^

### 5.6. Management of Patients with an Implantable Cardioverter-Defibrillator During Electrical Storm

Patients who develop ES in the presence of an ICD frequently receive multiple shocks. In addition to increasing stress and anxiety and being extremely painful, repetitive shocks enhance adrenergic activity, thereby perpetuating ventricular arrhythmias and increasing mortality. In some cases, patients arrive at the emergency department conscious, with stable blood pressure, while actively receiving shocks in the presence of the medical team. Placement of a magnet over the ICD generator disables shock therapies while the device remains within the magnetic field. This intervention may be useful to calm the patient and relieve pain, allowing adequate monitoring, appropriate sedation, IV antiarrhythmic therapy, oxygen support, and clinical stabilization, while avoiding shocks in an awake patient.

After the initial approach, prompt device interrogation is essential. Through telemetry, using the appropriate programmer for the specific ICD, it is possible to determine the cause of shocks (including whether they were inappropriate) and to identify the triggering mechanisms of ES. Polymorphic arrhythmias, MVT, and VF may have distinct modes of initiation, including short-coupled premature beats, pauses, or nonsustained VT. At this stage, modifications in detection settings, increases in the lower pacing rate, and even temporary deactivation of shock therapies should be considered.^68^

In some cases, LV epicardial pacing, the classic form of cardiac resynchronization therapy, may trigger ES due to heterogeneous dispersion of action potential duration or induction of reentrant VT within highly stable circuits. In such cases of ES induced by LV pacing in patients with biventricular ICDs, stabilization has been reported after disabling LV pacing.^69^

In patients with reentrant MVT and those with frequent episodes of nonsustained VT, programming longer detection times and enabling ATP therapies are effective strategies to reduce the number of shocks.^70^ Even in patients with ES and a high arrhythmic burden, a substantial proportion of VT episodes are self-terminating and do not require ICD intervention. Carefully tailored detection criteria, with sufficient delay to avoid shocks for nonsustained VT, improve patient comfort during ES. ATP therapies terminate a large proportion of VT episodes, including some cases in which tachycardia initially presents at very high rates within the VF zone.

ICD programming during ES should prioritize patient safety and minimization of harm. Increasing detection times, adjusting pacing rate and mode according to the individual clinical context, and favoring VT termination with ATP whenever possible are guiding principles of programming. In selected cases of recurrent shocks, immediate temporary deactivation with magnet placement over the ICD generator and/or device reprogramming to disable therapies may be necessary, provided that appropriate monitoring and supportive resources are available. Figure 7 presents a flowchart for ICD management in patients with ES.

**Figure 7:** Flowchart for emergency management and programming in patients receiving ICD therapies. ATP: antitachycardia pacing; ICD: implantable cardioverter-defibrillator.

## 6. Catheter Ablation in the Setting of Electrical Storm

Catheter ablation of VT is a critically important intervention in patients with ES, particularly when VT is monomorphic. After the initial clinical approach, patients who continue to experience recurrent VT episodes have an indication for emergency catheter ablation.^1,2,43,71-73^ Although these procedures are more complex, the ablation strategy is similar to catheter ablation of VT in other settings, involving VT mapping and substrate modification based on functional mapping. Patients with ES associated with BrS^74^ and VF^75,76^ may also undergo ablation; however, distinct techniques specific to these conditions are required.

Carbucicchio et al.^77^ prospectively investigated 95 patients with ischemic and nonischemic cardiomyopathy who underwent catheter ablation in the setting of ES refractory to antiarrhythmic drug therapy. In 62 patients, no VT was inducible (complete success); in 19, the clinical VT was no longer inducible (partial success), although nonclinical VT remained inducible; and in 14, the clinical VT remained inducible (failure). A second procedure was required in 12 patients and a third one in 6 patients. Among patients with complete or partial success, no recurrence of ES was observed. In contrast, among patients with failure after the final procedure, 8 of 10 (80%) experienced ES recurrence. During a follow-up of 22 months, 15 (16%) patients died, 11 (12%) from cardiac causes, including 4 sudden deaths; in all of these patients, ablation had been unsuccessful. Patients with partial success most frequently experienced VT recurrence during follow-up (65%), but without ES recurrence. Among patients with complete success, VT recurrence occurred in 16%. No procedure-related deaths were observed.

More recently, Muser et al.^78^ evaluated patients with ischemic and nonischemic cardiomyopathy and ES (n = 267). The procedural objective was elimination of the clinical VT and, when feasible, other mappable VTs. Over a median follow-up of 45 months, 76 (29%) patients died and 25 (9%) underwent heart transplantation. VT recurrence occurred in 87 (33%) patients, whereas ES recurrence was observed in only 13 (5%).

In patients with ES associated with VF, including during AMI, catheter ablation may also be considered. In such cases, the primary target is the premature ventricular complex that triggers VF. Ablation should be reserved for patients who remain refractory to optimized pharmacological therapy, with ischemia and hemodynamic support adequately optimized. The study by Komatsu et al.^76^ included 110 patients who developed ES due to VF after AMI. Most cases occurred during the acute (39%) and subacute (44%) phases of MI, corresponding to the first months after the event. ES was controlled in 92 patients (84%), and 30 (27%) died during hospitalization. Only 1 patient discharged from hospital experienced ES recurrence during a mean follow-up of 2.2 years. However, 29 (36%) patients died during the follow-up period.

A tool that can be used to estimate the risk of ES recurrence after ablation is the PAINESD score. Patients with a high score (> 15 points) may benefit from periprocedural hemodynamic support. The score includes:^1,2^

P – Chronic pulmonary disease – 5 points.

A – Age > 60 years – 3 points.

I – Prior AMI or ischemic cardiomyopathy – 6 points.

N – New York Heart Association functional class > III – 6 points.

E – LVEF < 25% – 3 points.

S – Prior cardiogenic shock – 9 points.

D – Diabetes mellitus – 3 points.

### 6.1. Additional Therapies for Electrical Storm

Sympathetic hyperactivity plays a fundamental role in the development and maintenance of arrhythmias. Increased adrenergic activity enhances automaticity, shortens the ventricular effective refractory period, and lowers the threshold for arrhythmia occurrence. Therefore, modulation of the cardiac–neural axis represents a therapeutic alternative for malignant ventricular arrhythmias, particularly ES.^29^

The efficacy of sympathetic block in controlling ES was demonstrated more than 20 years ago, with favorable effects of IV beta-blockers in reducing VF episodes and improving short-term survival.

In addition to beta-blockers, modulation of the cardiac–neural axis may be achieved through techniques such as sedation, thoracic epidural anesthesia, stellate ganglion block, surgical cardiac sympathetic denervation, and renal sympathetic denervation.

#### 6.1.1. Sedation

Sedation plays a central role in the therapeutic armamentarium for ES. The primary objectives of sedation in this context are to reduce the proarrhythmic effects of sympathetic tone, alleviate psychological stress, and achieve hemodynamic stabilization.

Sedation should be tailored to different clinical scenarios and may range from superficial sedation, aimed at pain relief and reduction of psychological distress, to deep sedation with orotracheal intubation and mechanical ventilation.

Deep sedation may be considered in cases of lack of response to antiarrhythmic therapy and multiple shocks occurring over a short interval. In this setting, sympathetic hyperactivity following repeated ICD therapies leads to clinical deterioration and triggers additional ventricular arrhythmias, contributing to a vicious cycle.^79^

The most commonly prescribed agents include benzodiazepines (e.g., midazolam), dexmedetomidine, and short-acting analgesics (e.g., fentanyl), which provide effective sedation without negative inotropic effects.

Propofol has several advantages that justify its use in cardiovascular settings, including rapid onset of action (2-3 minutes), short half-life, and amnestic properties. However, its narrow therapeutic window requires careful hemodynamic monitoring. Propofol is generally administered as a bolus followed by continuous infusion. Its main adverse effects include bradycardia, hypotension, and respiratory depression. Although rare, cardiogenic shock may occur due to its negative inotropic effect.^80^

In addition to reducing sympathetic activity and increasing vagal tone, propofol alters baroreflex sensitivity and atrioventricular conduction. Moreover, propofol acts directly on cardiomyocytes through changes in protein kinase C translocation to different intracellular targets.

A study involving 15 patients with ES refractory to initial treatment who underwent deep sedation (primarily with propofol and concomitant or subsequent infusion of other agents, including fentanyl) with orotracheal intubation demonstrated complete resolution of VT in 80% of cases and partial resolution in 13%.^81^

In a multileft retrospective study conducted by Martins et al., the effectiveness of deep sedation was evaluated in 116 patients with ES refractory to antiarrhythmics. Rapid-acting hypnotic agents and neuromuscular blockers were used for induction and intubation, followed by administration of an IV hypnotic agent (midazolam or propofol) and opioids for maintenance. An acute response, observed within 15 minutes, occurred in nearly half of the cases and was an independent predictor of improved in-hospital survival, being associated with a 55% lower risk of death.^82^

Dexmedetomidine stands out for its lower risk of respiratory depression, as well as its anxiolytic effect and sympathetic suppression. Case reports suggest that it may reduce the frequency of ICD shocks and VT recurrence and may be useful even in unstable patients.^2^

#### 6.1.2. Thoracic Epidural Anesthesia

Thoracic epidural anesthesia consists of infusion of a long-acting local anesthetic, such as bupivacaine or ropivacaine, into the T1-T2 or T2-T3 epidural space. The procedure may be performed at the bedside or with fluoroscopic guidance. Thoracic epidural anesthesia is an effective therapy with rapid onset and short duration that may be considered in cases of incessant, refractory VT. However, this technique is contraindicated in patients requiring dual antiplatelet therapy or anticoagulation.^83^ The proposed antiarrhythmic mechanism involves reduction of cardiac sympathetic tone, with consequent prolongation of action potential duration and refractory period.^84^

Available data on this topic are limited. Bourke et al. demonstrated an 80% reduction in ventricular arrhythmia burden in 75% of patients who were nonresponsive to ablation and underwent thoracic epidural anesthesia.^85^ In another report, 45% of patients showed favorable responses.^41^

From a practical perspective, the favorable safety profile and low rates of hypotension make thoracic epidural anesthesia a promising alternative to prolonged deep sedation with orotracheal intubation in patients who are unresponsive to antiarrhythmics and/or ablation.^41^

#### 6.1.3. Stellate Ganglion Block

Stellate ganglion block is a bedside procedure performed under fluoroscopic guidance or, preferably, ultrasound guidance. It involves local injection of an anesthetic agent (bupivacaine or ropivacaine) into the left stellate ganglion, with the aim of temporarily reducing adrenergic activity by blocking afferent and efferent neural pathways. This technique may serve as an alternative in patients refractory to percutaneous ablation or as a temporary measure until definitive adrenergic blockade strategies are established. Bilateral block should be reserved for intubated patients because of the risk of phrenic nerve paralysis with respiratory compromise. One study demonstrated a 93% reduction in acute ventricular arrhythmia occurrence and a 90% reduction in arrhythmic burden in 56% of patients during a follow-up longer than 3 years when left stellate ganglion block was combined with cardiac sympathetic denervation.^86^

A case series,^87^ a randomized study,^29^ and meta-analyses have also shown benefits of this procedure in the temporary suppression of ventricular arrhythmias,^42,88^ with satisfactory safety and effectiveness.

#### 6.1.4. Surgical Cardiac Sympathetic Denervation

Surgical cardiac sympathetic denervation provides permanent neuraxial blockade of cardiac afferent and efferent innervation. In 2014, Vaseghi et al. evaluated the effects of cardiac sympathetic denervation in 41 patients with refractory ventricular arrhythmias or ES and demonstrated significant reductions in the number of ICD shocks over the medium and long term with both unilateral and bilateral denervation, with more favorable outcomes observed with bilateral denervation.^89^ Late adverse effects (> 3 months after the procedure) included sensory changes, altered sweating, and ptosis.

The procedure is usually performed via video-assisted thoracoscopic surgery and involves resection of part of the stellate ganglion and the second to fourth thoracic ganglia. The benefits of this intervention have been described in patients with LQTS and catecholaminergic PVT (CPVT).^90^

#### 6.1.5. Renal Sympathetic Denervation

Percutaneous renal denervation is a selective, minimally invasive endovascular approach associated with low complication rates and no reported systemic adverse events. The procedure involves ablation of the neural plexus within the adventitia of the renal artery, thereby reducing central sympathetic outflow to the heart.

Several studies have demonstrated the benefits of renal denervation in the treatment of ES, including reductions in the number of ventricular arrhythmia episodes and appropriate ICD therapies in patients with ventricular arrhythmias refractory to optimized antiarrhythmic drug therapy and ablation. The procedure has been shown to be safe and was not associated with significant adverse events or changes in blood pressure or renal function.^91^ Experience with renal denervation in patients with Chagas disease is limited and derives from a single study demonstrating the feasibility and safety of this procedure in reducing ICD therapies; it may be considered in refractory cases or when catheter ablation is contraindicated.^92^

#### 6.1.6. Circulatory Management

In unstable patients with hemodynamic compromise and recurrent episodes of VT/VF with syncope, mechanical circulatory support with a LV assist device or extracorporeal membrane oxygenation (ECMO) may be considered as a bridge or supportive strategy for catheter ablation or heart transplantation.^93^

Among the available devices are ECMO, the TandemHeart circulatory support system, the intra-aortic balloon pump (IABP), and the Impella device.

#### 6.1.7. Extracorporeal Membrane Oxygenation

In addition to ensuring tissue perfusion and reducing LV end-diastolic pressure, venoarterial ECMO provides additional benefits, including the ability to achieve adequate antiarrhythmic levels and to facilitate rapid weaning from catecholamines.^93^ These combined effects contribute to satisfactory restoration and maintenance of sinus rhythm. Le Pennec-Prigent et al. reported restoration of sinus rhythm after 3 hours in 61.5% of 26 patients with refractory ES and cardiogenic shock treated with ECMO.^94^

Complications, high costs, and elevated mortality rates in patients with refractory VT limit the use of venoarterial ECMO to specialized lefts.^95^

#### 6.1.8. Intra-Aortic Balloon Pump

The effectiveness of the IABP in the treatment of refractory arrhythmias in patients with coronary artery disease is well recognized; however, data in noncoronary patients are limited. Its low cost, wide availability, ease of use, and low invasiveness make the IABP a tool to be considered in situations requiring low- to moderate-level LV support.^96^

#### 6.1.9. TandemHeart Circulatory Support and Impella Device

Several advantages have been demonstrated with the use of ventricular assist devices such as Impella and TandemHeart. Impella reduces filling pressures and afterload, resulting in significant improvement in myocardial perfusion and providing cardioprotective effects.^97^ However, Impella has limitations in providing adequate hemodynamic support in cases of unstable VT.

#### 6.1.10. Device Selection

Device selection depends on disease severity, individual patient characteristics, and the availability of the technical and human resources required for appropriate management. Prophylactic use of percutaneous ventricular assist devices in patients at high risk of hemodynamic compromise during ES ablation yields better outcomes than rescue use. In a study conducted by Muser et al., preventive use of these devices was associated with reduced hemodynamic decompensation during ablation as well as lower mortality and reduced need for heart transplantation.^98^ In the study by Mathuria et al., rescue use of circulatory support devices during ablation was associated with high 30-day mortality rates.^99^

A systematic review including 2,465 patients demonstrated reduced mortality rates with prophylactic mechanical circulatory support as an adjunctive strategy in patients with ES and high-risk PAINESD scores. In contrast, rescue mechanical circulatory support during ablation was associated with high mortality rates.^100^

## 7. Management of Electrical Storm Associated with Channelopathies

In patients without structural heart disease, ES may be associated with channelopathies. The main channelopathies include BrS, LQTS or SQTS, and CPVT. Other conditions have less well-defined pathophysiology but exhibit behavior similar to channelopathies, with the potential to cause rapid PVT, TdP, and VF even in the absence of significant structural heart disease. These include Haïssaguerre syndrome (also referred to as early repolarization syndrome [ERS]) and TdP triggered by ultra-short-coupled premature ventricular complexes (TdP-USC), a form of PVT.

A specific therapeutic target can be identified in patients with an established diagnosis. However, when ES is the first manifestation of the condition, arrhythmia triggers (e.g., exercise, emotional stress, and intoxication) may help guide additional therapeutic strategies beyond basic measures such as correction of electrolyte disturbances.

In channelopathy-related ES, as in cases of ES in the broader sense, three core principles should also be considered, namely: i) control of triggering factors (electrolyte disturbances, proarrhythmia, and related contributors); ii) control of the arrhythmia itself, including ICD-specific reprogramming, pharmacological therapy, and/or catheter ablation; and iii) autonomic modulation (beta-blockers, sympathetic denervation, and sedation)^101^ (Figures 8 e 9).

**Figure 8:** Management of electrical storm in channelopathies. aLQTS: acquired long QT syndrome; BB: IV beta-blockers; BrS: Brugada syndrome; CCB: calcium channel blockers (verapamil); cLQTS: congenital LQTS; CPVT: catecholaminergic polymorphic ventricular tachycardia; ERS: early repolarization syndrome; HR: heart rate; ICD: implantable cardioverter-defibrillator; IPR: isoproterenol; LCSD: center cardiac sympathetic denervation; Mg/K: potassium and magnesium replacement; OTI: orotracheal intubation; PM: pacemaker; PM ≥ 70: PM programmed with a HR ≥ 70 bpm; QD: quinidine; RFA: radiofrequency ablation; TdP-USC: *torsades de pointes* triggered by ultra-short-coupled premature ventricular complexes; VF: ventricular fibrillation.

**Figure 9:** The 10 most important messages in the management of electrical storm in patients with channelopathies. ACLS: Advanced Cardiovascular Life Support; aLQTS: acquired long QT syndrome; CEATOX: Poison Control Center (Centro de Assistência Toxicológica); ICD: implantable cardioverter-defibrillator; IV: intravenous.

IV magnesium is frequently used in the management of ES. It is an essential transmembrane and intracellular modulator of the electrical activity of cardiac cells. It can be administered to patients with either hypomagnesemia or normomagnesemia. Magnesium is generally well tolerated, even in hemodynamically unstable situations; moreover, its administration is rapid and straightforward. Magnesium is the treatment of choice for patients with TdP-type tachycardia, long QT interval, and digitalis-induced cardiac arrhythmias.^102^

Increased sympathetic tone plays a fundamental role in the development and maintenance of many ventricular arrhythmias.^103^ A vicious cycle may occur in ES, particularly in patients with an ICD. Shocks can trigger sympathetic discharge and lead to additional ventricular arrhythmias and further shocks. This process can continue indefinitely.^104^ Nonselective beta-blockers, such as propranolol, are frequently used in adrenergic-dependent channelopathies, including CPVT and congenital LQTS (cLQTS). In contrast, arrhythmia onset in certain conditions, such as BrS, ERS, SQTS, and TdP-USC, usually occurs during sleep or at rest, situations characterized by increased parasympathetic tone. The use of isoproterenol in such contexts may be effective in preventing arrhythmia recurrence.^105^

Management of ES also includes guidance on interventions that should be avoided. For this purpose, specific websites are available for consultation regarding contraindicated medications in BrS and LQTS, namely brugadadrugs.org and crediblemeds.org, respectively (Figure 9). As lidocaine shortens the QTc interval, it should also be avoided in SQTS.

In patients with BrS, incessant arrhythmia may be treated with IV isoproterenol infusion,^106^ followed by oral quinidine (or cilostazol if refractory to quinidine),^105^ although the use of quinidine is limited by lack of availability in large parts of the world.^107^ Oral sotalol may be an option, provided that QTc interval monitoring and careful assessment of individual patient response are ensured.^108^ Catheter ablation of the epicardial RV outflow tract, targeting low-voltage scar areas and/or regions of slow conduction (or those unmasked by IV ajmaline), has been shown to be highly effective in BrS.^74^

Similarly, ERS, although still lacking a well-defined pathophysiological mechanism, shares similarities with BrS and guides the management of ES toward the same rationale, namely IV isoproterenol infusion followed by quinidine. Specifically in BrS, multiple inappropriate shocks may be triggered by T-wave oversensing and should be promptly corrected through ICD reprogramming.^109^

Isoproterenol may also be used to increase HR in patients with acquired LQTS as a bridge to pacemaker implantation. Its effects include HR acceleration through nonselective beta1/beta2-adrenergic agonism, which leads to QT interval shortening and reduction of the effective refractory period when potassium channels are functionally preserved. In contrast, in patients with cLQTS, in whom potassium channel function is impaired, isoproterenol prolongs the QT interval and induces early afterdepolarizations, increasing repolarization dispersion and, consequently, promoting ES.^110^

In cLQTS, in addition to IV magnesium administration and oral propranolol, when feasible, patients may require temporary pacing to maintain an increased HR and suppress premature beats. In selected cases, IV lidocaine, administered as bolus followed by continuous infusion, may be required for maintenance therapy. Importantly, when pacing is used, the minimum pacing rate should be set above 80 bpm to avoid pause-dependent malignant arrhythmias, such as those observed in type 2 cLQTS or medication-induced forms.^111^

In the rare SQTS, initial management may include IV isoproterenol,^112^ and quinidine may also be considered,^113^ despite its limited availability in Brazil. Although robust evidence is lacking due to the rarity of this condition, case reports suggest potential benefit of high-dose quinidine in selected scenarios.^114^

In bidirectional or PVT, characteristic of CPVT, beta-blockers are the treatment of choice. Sedation may serve as a useful adjunct in emergency settings, as may oral propranolol and propafenone. If treatment is insufficient or poorly tolerated for sympathetic suppression, selected patients may benefit from autonomic modulation, including percutaneous stellate ganglion block, thoracic epidural anesthesia, or left cardiac sympathetic denervation.^115^

Some patients, regardless of the underlying channelopathy, may present with premature ventricular complexes, particularly those originating from the Purkinje system (moderator band of the RV, anterior or posterior fascicle of the LV), the LV outflow tract, the tricuspid annulus, or the papillary muscles, which can trigger ES. In such cases, catheter ablation may be directed at the triggering ectopic focus. A peculiar variant of ES is TdP-USC, a subtype of PVT. Its pathophysiology remains unknown, and it should not be considered a channelopathy. This condition may be identified by documentation of TdP, even in the absence of ischemia, structural heart disease, or QT interval prolongation. In this rare scenario, high-dose verapamil (mean doses up to 480 mg/day) is also indicated.^116^

Table 3 summarizes the main recommendations regarding pharmacological selection and pacemaker use in emergency settings.

**Table 3:** Intravenous medications and PM in the treatment of electrical storm associated with channelopathies

	Yes	No
Magnesium	cLQTS, TdP-USC	—
Isoproterenol	BrS, ERS, aLQTS, SQTS	cLQTS
Verapamil	TdP-USC	—
Lidocaine	cLQTS	SQTS?
Esmolol	CPVT	—
Amiodarone	SQTS?	cLQTS, aLQTS, BrS, CPVT
PM	cLQTS, aLQTS	—

aLQTS: acquired long QT syndrome; BrS: Brugada syndrome; cLQTS: congenital LQTS; CPVT: catecholaminergic polymorphic ventricular tachycardia; ERS: early repolarization syndrome; SQTS: short QT syndrome; TdP-USC: torsades de pointes triggered by ultra-short–coupled premature ventricular complexes; PM: temporary pacemaker with a pacing rate > 80 bpm.

Oral maintenance medications specific to each channelopathy should be initiated or reinitiated early, such as mexiletine in cLQTS type 3, nadolol or propranolol in cLQTS types 1 and 2, sotalol in BrS (when quinidine is unavailable) or in SQTS, and propafenone in CPVT.^101^

## 8. Long-Term Prevention of Recurrence

The occurrence of ES is associated with increased mortality both in the acute phase and over the long term.^2^ Therefore, after initial patient stabilization, measures aimed at preventing new events and reducing long-term recurrence should be considered.

### 8.1. Pharmacotherapy

Optimized medical treatment of the underlying cardiac disease is essential for preventing ES episodes. In patients with HF with reduced ejection fraction, the recommendations of the Brazilian Guidelines for Chronic and Acute Heart Failure include treatment with angiotensin-converting enzyme inhibitors, angiotensin II receptor blockers, or angiotensin receptor-neprilysin inhibitors, mineralocorticoid receptor antagonists, beta-blockers, and sodium-glucose cotransporter 2 inhibitors, with the goal of reducing HF-related mortality and sudden cardiac death.^117^ Coronary angiography should be considered in patients presenting with chest pain, ST-segment changes, or significant elevations in troponin levels.

Antiarrhythmics, in addition to their use for acute stabilization of ES, may also be considered for the prevention of recurrence. In a randomized clinical trial, the combination of amiodarone and a beta-blocker was superior to beta-blocker monotherapy or sotalol in reducing ICD shocks.^118^ However, a significant rate of treatment discontinuation due to adverse effects was observed. In selected and less frequent scenarios, specific medications may be indicated for ES prevention, such as mexiletine in cLQTS type 3 and quinidine in BrS.^2^

In patients with a history of ES, strict vigilance is required regarding the use of any medication with potential proarrhythmic effects or the ability to induce metabolic or electrolyte disturbances that may trigger ES.

### 8.2. Implantable Cardioverter-Defibrillator

ES may represent the initial clinical manifestation of ventricular arrhythmia. In patients with ES associated with hemodynamic instability or resuscitated cardiac arrest, and in the absence of a reversible cause, ICD implantation is indicated as a strategy for secondary prevention.^59^ Meta-analysis data show that ICD implantation in these patients is associated with reductions in both arrhythmic and all-cause mortality.^119^ When evaluating ICD indication, comorbidities, quality of life, and life expectancy should be carefully considered. Importantly, in the setting of ES, ICD implantation should not be performed while incessant arrhythmia persists without adequate clinical control.

### 8.3. Catheter Ablation

Catheter ablation may be considered both for acute stabilization of patients with ES and for long-term prevention of recurrence.

In patients with ischemic heart disease and an ICD, randomized clinical trials have demonstrated that catheter ablation is superior to antiarrhythmic drug therapy alone in preventing new ICD therapies.^120-123^

Registry data indicate that patients with ES referred for ablation are older, have lower ejection fraction, and exhibit a higher prevalence of comorbidities compared with patients without ES.^73^ Regarding ablation outcomes in patients with ES, registry data show a VT-free survival at 60 months of 54%, with no significant difference between ischemic and nonischemic etiologies.^73,78^ Acute procedural success, defined as noninducibility of ventricular tachyarrhythmias, was associated with improved survival. Complication rates were in the 3%-7% range.^12,14^ In registries with smaller sample sizes, ablation has also proven effective in patients with ES and arrhythmogenic RV cardiomyopathy or Chagas disease.^124^

### 8.4. Autonomic Modulation

Autonomic modulation may be considered for both acute stabilization and long-term prevention of recurrence in patients with ES. Left or bilateral cardiac sympathectomy has been shown, in case series, to significantly reduce the recurrence of ICD shocks.^16^

## 9. New perspectives

ES is a life-threatening medical emergency with a poor prognosis. Its therapeutic management is challenging and largely based on retrospective studies, underscoring the urgent need for novel treatment strategies. One factor contributing to the difficulty in managing ES is the broad spectrum of underlying cardiac conditions. ES may occur in association with structural heart disease, genetic arrhythmic syndromes, or even in patients with structurally normal hearts. This heterogeneity results in diverse electrophysiological characteristics and distinct arrhythmic mechanisms. Nevertheless, a common feature across these scenarios is the critical role of the autonomic nervous system in the initiation and maintenance of ventricular arrhythmias. The TEMPEST study^18^ demonstrated that ES occurs predominantly during working hours and on working days, suggesting a circadian variation in its distribution and supporting the role of sympathetic tone in its occurrence.

In order to counteract the hyperadrenergic state, sedation with opioids or hypnotic agents, such as midazolam or propofol, may contribute to a reduction in arrhythmic events. In a study involving 15 patients during ES, deep sedation with propofol successfully terminated ventricular arrhythmias in 80% of cases.^81^

Neuromodulation represents an emerging therapeutic approach for ventricular arrhythmias. Cardiac sympathetic denervation has been shown to reduce the recurrence of VT and ICD shocks in patients with ES in both the short and long term. At 1 year, 58% of patients remained free from shocks and experienced no recurrence of ventricular arrhythmia.^125^ Other forms of autonomic modulation, such as thoracic epidural anesthesia and renal denervation, are currently under investigation and have yielded promising results.^41,126^

For unstable patients who do not respond to conventional therapy, ECMO may be used as a bridge until a more definitive treatment can be implemented. However, the reported success rate of this strategy is approximately 50%, indicating that new treatment algorithms should be explored.^94^

Finally, stereotactic ablation is emerging as an alternative modality for patients with ES. Stereotactic body radiotherapy is routinely used in oncology for the noninvasive treatment of solid tumors with high precision and efficacy. It is currently being tested in cases of ventricular arrhythmias, with promising results demonstrating an approximately 70% reduction in VT recurrence. However, its role in ES is more limited due to the absence of an immediate therapeutic effect.^127-130^ The success of this modality has been attributed to radiation-induced fibrosis,^131^ which creates a conduction block within the myocardium. Nonetheless, the reduction in ventricular arrhythmias typically occurs several days after the procedure, suggesting that additional mechanisms may also be involved.^132^

## Data Availability

The data underlying the text of the Position Statement are included in the manuscript.
